# Therapeutic potential and pharmacological insights of total glucosides of paeony in dermatologic diseases: a comprehensive review

**DOI:** 10.3389/fphar.2024.1423717

**Published:** 2025-01-02

**Authors:** Huige Wang, Wenchao Yu, Tong Wang, Dianwei Fang, Zeyun Wang, Yuanhong Wang

**Affiliations:** ^1^ Heilongjiang University of Chinese Medicine, Harbin, China; ^2^ Department of Dermatology, The First Affiliated Hospital of Heilongjiang University of Chinese Medicine, Harbin, China

**Keywords:** total glucosides of paeony, dermatologic diseases, immunomodulatory, mechanism of action, Chinese herbal medicine

## Abstract

Total glucosides of paeony (TGP) are a group of monoterpenes extracted from *Paeonia lactiflora Pall.*, primarily including metabolites such as paeoniflorin and oxypaeoniflorin. Modern pharmacological studies have shown that TGP possesses a variety of biological effects, including immunomodulatory, anti-inflammatory, hepatoprotective, nephroprotective, antidepressant, and cell proliferation regulatory activities. In recent years, clinical research has demonstrated favorable therapeutic effects of TGP on disorders of the liver, cardiovascular, nervous, endocrine, and skeletal systems. Particularly in dermatological treatments, TGP has been found to significantly improve clinical symptoms and shorten the course of the disease. However, there are still certain limitations in the scientific rigor of existing studies and in its clinical application. To assess the potential of TGP in treating dermatologic diseases, this article provides a review of its botanical sources, preparation and extraction processes, quality control, and major chemical metabolites, as well as its pharmacological research and clinical applications in dermatology. Additionally, the mechanisms of action, research gaps, and future directions for TGP in the treatment of dermatologic diseases are discussed, offering valuable guidance for future clinical research on TGP in dermatology.

## 1 Introduction

The skin, being the largest organ of the human body, covers the entire surface of the body and functions as a crucial protective barrier (Wong et al., 2023). However, the skin is susceptible to microbial, chemical, physical, and immunologic factors that can cause dermatologic diseases (Mummed et al., 2018). Dermatologic disease is a general term for diseases that occur in the skin and its accessory organs (Lim et al., 2022). Interestingly, a variety of diseases occurring in internal organs can also manifest in the skin (Valdez et al., 2015). At present, there are over 2,000 recognized dermatologic diseases with unique clinical characteristics. This extensive variety is indicative of the diverse nature of these conditions and the challenges in their treatment and diagnoses. The incidence of dermatologic diseases has surged due to a confluence of various factors. Social progress, changes in the living environment, increasingly serious air pollution, and poor living and eating habits collectively contribute to this increase. According to a report by the World Health Organization (WHO), approximately 20%–30% of the global population suffers from some form of dermatologic disease (Gu et al., 2024). This means that over 1.5 to 2 billion people worldwide are currently dealing with skin health issues. These conditions can affect individuals at any age, from birth to death, and can involve any part of the body. Some dermatologic diseases are difficult to cure and are prone to recurrence even after treatment. The health burden caused by dermatologic diseases ranks fourth among all diseases in terms of disability-adjusted life years. This not only significantly impacts patients’ quality of life but also places a substantial strain on global healthcare systems (Adly et al., 2021; Donetti et al., 2023).

Clinical manifestations associated with dermatosis often involve sensations of itchiness, pain, and numbness because the skin tissue is very rich in nerves. Beyond physical symptoms, dermatologic injuries can lead to secondary psychological problems such as anxiety, anger, the contagious nature of certain dermatologic diseases poses not only a threat to individual wellbeing but also causes widespread panic and social discrimination, causing great physical and psychological pain to patients (Feldo et al., 2022). Clinically, antibiotics, antihistamines, painkillers, or hormonal drugs are commonly used to treat dermatologic diseases (Fattah and Darwish, 2013). However, these therapeutic measures are associated with certain limitations. The efficacy of these medications is short-lived, and the dermatologic disease is prone to relapse after cure (Xie Y. X. et al., 2023). A major issue is the emergence of drug resistance resulting from prolonged usage of these medications. Additionally, researches have noted potential negative impacts of these medications on liver and kidney functionality, as well as the nervous system. Therefore, the use of these medications is restricted in certain populations, such as pregnant women, the elderly, and individuals in specific high-risk occupations, including pilots, athletes, law enforcement officers, and military personnel, due to their potential adverse effects.

Recently, important insights have emerged about the crucial function of natural phytochemicals in the clinical management of dermatologic disease, such as green tea polyphenols, apigenin, shikonin and others (Fowler et al., 2010; Ranneh et al., 2016; Pourang et al., 2020; Xie W. et al., 2023). These natural medicines present distinct advantages, being less irritating to the skin, less prone to inducing resistance, and exhibiting a good protective effect on the microbiome balance of the skin (Cintosun et al., 2020; Patel et al., 2023). Zhu et al. utilized Epigallocatechin-3-gallate (EGCG), the main chemical metabolite of green tea polyphenols, to treat vitiligo induced by topical application of monobenzone in a mouse model. Histological examination suggested a reduction in CD8^+^ T cell infiltration at the lesions, and it was hypothesized that EGCG might have an inhibitory effect on vitiligo autoimmunity (Zhu et al., 2014). Zhang et al. conducted research wherein PIG3V cells, stimulated with H_2_O_2_, were treated with apigenin (a plant-derived aglycone). Subsequently, they assessed the activity and oxidative stress-related parameters by enzyme-linked immunosorbent assay. Their findings indicated that apigenin enhanced the expression of cellular antioxidants superoxide dismutase (SOD), catalase (CAT), and glutathione peroxidase (GSH-Px) and shielded melanocytes from oxidative damage through activation of the Nrf2 pathway (Zhang et al., 2020). Wu et al. found that the activity of the human oncogene p53 was enhanced in a cycle-blocking assay involving shikonin, a naphthoquinone metabolite extracted from *Arnebiae Radix*, on human malignant melanoma cells. This study demonstrated that shikonin inhibited the growth of A375-S2 cells in a time- and concentration-dependent manner, with a 48-hour IC50 value of 7.1 ± 0.9 μmol/L (Wu et al., 2004). Another study reported that shikonin derivatives induced apoptosis in mouse melanoma high metastatic cells B16F10 by activating caspase3, a protease involved in apoptosis execution. Furthermore, these derivatives induced sub-G1 cell cycle arrest, showing anticancer activity (Rajasekar et al., 2012). In addition, a significant finding was made about the effects of SSA (saikosaponin A) and SSC (saikosaponin C) obtained from *Radix bupleuri*. It was discovered that these substances inhibited TNF-α-induced thymic stromal lymphopoietin (TSLP) expression at the transcriptional level through the suppression of mitogen-activated protein kinase (MAPK) signaling, which mediates the transcriptional activation of early growth response factor-1 (EGR-1) in human immortalized epidermal cells (HaCaT) keratinocytes. The topical use of SSA or SSC was shown to enhance the condition of atopic dermatitis-like skin lesions in 2, 4- dinitrochlorobenzene (DNCB) mice (Ahn et al., 2022). These studies indicate that natural phytochemicals significantly affect the clinical management of dermatologic conditions, highlighting their potential, research importance, and practical value in treating dermatologic diseases.

Among the natural phytochemicals, the total glucosides of paeony (TGP), extracted from *Paeonia lactiflora Pall.*, have been widely applied in clinical research for the treatment of dermatologic diseases and have garnered significant attention from scholars. To further elucidate the mechanisms of action and potential applications of TGP in dermatologic diseases, this article provides an overview of the botany and traditional uses of *Paeonia lactiflora Pall.*. Additionally, it reviews the extraction and purification processes of TGP, its main active metabolites, quality control measures, as well as clinical research and pharmacological mechanisms related to its use in dermatologic diseases. Furthermore, the article identifies gaps in current research and suggests directions for future studies. By reviewing these aspects, the article seeks to provide a valuable and comprehensive reference for future in-depth clinical investigations.

## 2 Materials and methods

The literature review was performed using various databases, including Web of Science (https://webofscience.clarivate.cn), Baidu Scholar (http://xueshu.baidu.com/), ScienceDirect (www.sciencedirect.com), PubMed (https://www.ncbi.nlm.nih.gov/), CNKI (https://www.cnki.net/), and WanFang DATA (http://www.wanfangdata.com.cn/index.html). Additional resources were obtained from library searches encompassing classical texts on Chinese herbal medicine, peer-reviewed journals, local periodicals, and master’s and doctoral dissertations. The search utilized keywords such as “Total glucosides of paeony,” “*Paeonia lactiflora Pall*.,” “*Paeonia veitchii Lynch*,” “*Paeoniae Radix Alba* or white peony,” “Paeoniflorin,” “Albiflorin,” “TGP,”“dermatologic disease,”“skin disease,” and their combinations. Duplicate articles identified during the search process were excluded. The Plant List database (http://www.plantsoftheworldonline.org/) was consulted to confirm scientific names and provide details on subspecies and varieties of Paeonia plants.

## 3 Botanical description and distribution of *Paeonia lactiflora Pall.*

**FIGURE 1:**
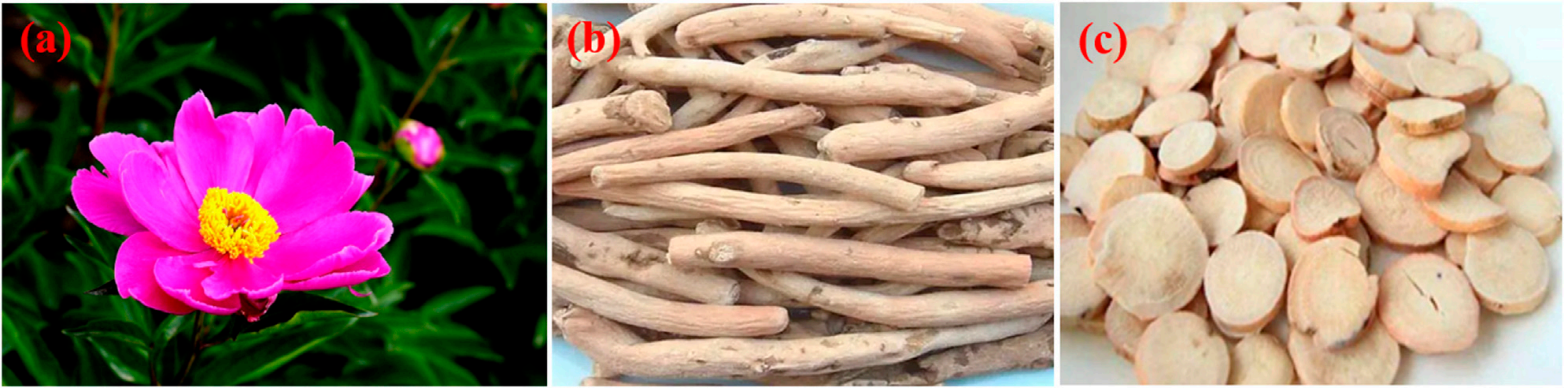
Photographs of above-ground parts **(A)**, medicinal portion **(B)** and commercially applied botanical drugs of *Paeonia lactiflora Pall.* **(C)**.

*Paeonia lactiflora Pall.* is a perennial herbaceous plant belonging to the *Ranunculaceae* family and the *Paeoniaceae* subfamily. There are approximately 30 species of plants in the *Paeonia* genus, which are mainly divided into two categories: herbaceous peonies and tree peonies. Native to China, it is widely distributed across the temperate regions of Asia, North America and Europe. The plant features robust rhizomes and compound leaves with a deep green coloration, and the leaf margins often exhibit wavy or serrated edges. *Paeonia lactiflora Pall.* is renowned for its large, vibrant flowers, which come in a variety of colors, including white, pink, red and purple, making it highly valued for ornamental purposes (Figures 1A). The rhizome of *Paeonia lactiflora Pall.* holds significant medicinal value in traditional Chinese medicine (Xie et al., 2017; Zhang et al., 2022a). According to the 2020 edition of the Pharmacopoeia of China, the roots of *Paeonia lactiflora Pall.* are harvested in the summer and autumn seasons. After harvesting, the roots are cleaned, boiled, peeled and then sun-dried. The dried root of the plant *Paeonia lactiflora Pall* (Figures 1B, C). has been used in traditional Chinese medicine for many centuries.

## 4 Traditional uses of *Paeonia lactiflora Pall.*

The medicinal history of *Paeonia lactiflora Pall.* (Shao Yao) can be traced back to the ''Shen Nong Ben Cao Jing'', the earliest extant pharmacopeia in China, written during the Dong Han dynasty (approximately A.D. 25–220). In the ''Shen Nong Ben Cao Jing'', Shao Yao classified as a top-grade medicinal botanical drug with nourishing properties. The book provides a detailed description of its medicinal properties and therapeutic effects, stating that it can nourish the blood, soothe the liver and alleviate pain and muscle spasms. In China, hundreds of ancient formulas have used *Paeonia lactiflora Pall.* as the main drug, such as Danggui Shaoyao Powder, Zhishi Shaoyao Powder, Baizhu Shaoyao Powder, Shaoyao Decoction, and Shaoyao Gancao Decoction (Tian et al., 2022). Therefore, *Paeonia lactiflora Pall.* is one of the most important botanical drugs in traditional medicine.

## 5 The extraction and purification process of TGP

The dried roots of *Paeonia lactiflora Pall.*, conforming to pharmacopoeial standards, are selected as raw materials and pulverized to a fine particle size. Water and ethanol are commonly used as extraction solvents, employing techniques such as maceration, reflux extraction, and ultrasound-assisted extraction (Wu et al., 2019). However, the crude extract of TGP typically contains a significant number of impurities. Therefore, purification is necessary to obtain high-purity TGP. The primary methods for the isolation and purification of TGP currently include filtration and rotary evaporation, organic solvent extraction, column chromatography, and recrystallization (Deng et al., 2010; Peng et al., 2023).

## 6 Main active metabolites of TGP

TGP primarily contains various monoterpene glycosides. (Chen et al., 2020; Xu et al., 2023). The key metabolites include paeoniflorin (PF), albiflorin, oxypaeoniflorin, benzoylpaeoniflorin, benzoyloxypaeoniflorin, lactiflorin, albiflorin R1, and benzoylpaeoniflorin lactone. Notably, PF accounts for more than 90% of the TGP (Santana et al., 2016; Tu et al., 2019; Xu et al., 2023). As the primary active metabolite of *Paeonia lactiflora Pall.*, PF essentially represents the main efficacy of TGP (Zhang et al., 2008; Jin and Zhang, 2022; Liu et al., 2023). The main monoterpene glycosides metabolites of TGP are shown in Table 1.

**TABLE 1:** The main chemical metabolites of the monoterpene glycosides in TGP.

PubChem CID	Metabolites	Molecular formula	Molecular weight	Molecular structure
442,534	Paeoniflorin	C_23_H_28_O_11_	480.5	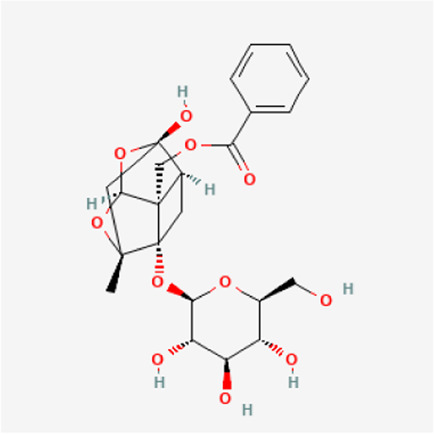
51,346,141	Albiflorin	C_23_H_28_O_11_	480.5	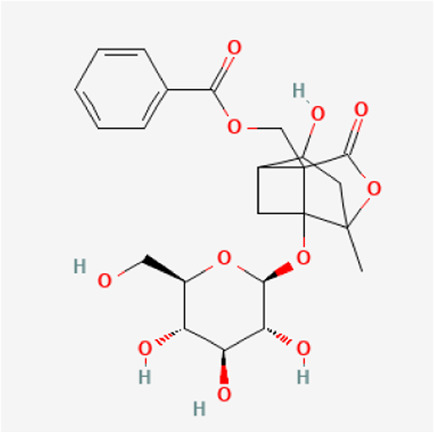
21,631,105	Oxypaeoniflorin	C_23_H_28_O_12_	496.5	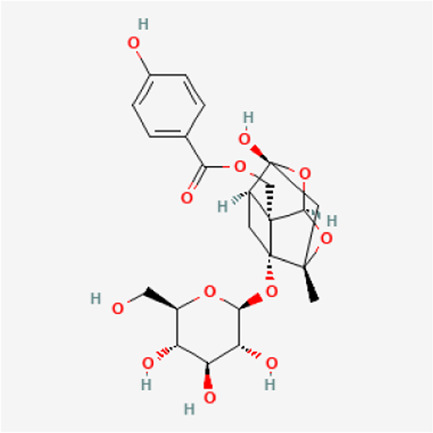
21,631,106	Benzoylpaeoniflorin	C_30_H_32_O_12_	584.6	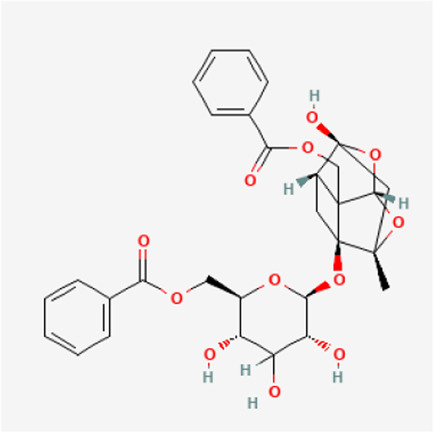
138,113,866	Benzoyloxypeoniflorin	C_30_H_32_O_13_	600.6	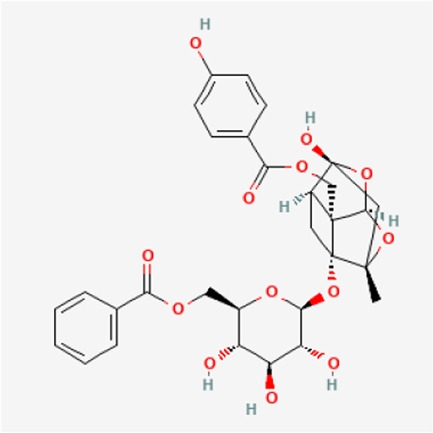
14,605,198	Lactiflorin	C_23_H_26_O_10_	462.4	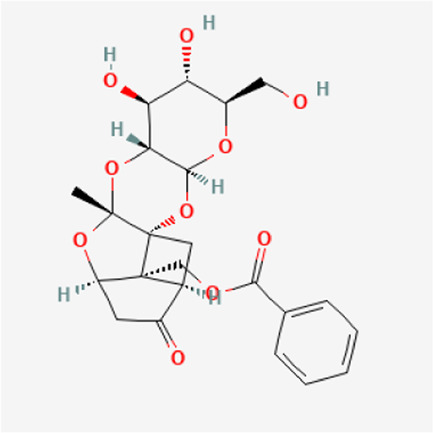
50,163,461	Albiflorin R1	C_23_H_28_O_11_	480.5	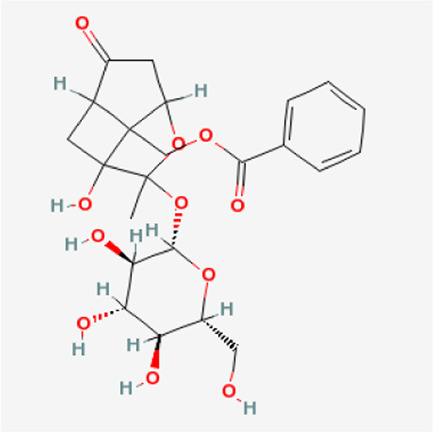
102,000,323	Benzoylalbiflorin	C_30_H_32_O_12_	584.6	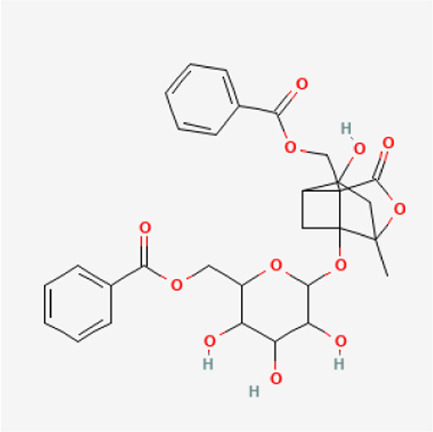

## 7 Phytochemical quality control of TGP

The quality of herbal products used by humans is of paramount importance due to their direct impact on human health. Moreover, with the rapidly increasing demand for traditional herbal products in recent years, it is essential to implement stringent quality control measures (Jakimiuk and Tomczyk, 2024). The WHO established guidelines for quality control of medicinal plant materials, focusing on factors such as organoleptic properties, ash content, moisture levels, microbial contamination, and chromatographic and spectroscopic analysis (Chandel et al., 2011). The qualitative assessment of herbal products is primarily conducted using UV, IR and TLC techniques, while quantitative analysis is typically performed using methods such as HP-TLC, HPLC, SFC, LC-MS or GC-MS (Ramaswamy et al., 2014). From a pharmacological perspective, *Paeonia lactiflora Pall.* seems to be the most valuable. The quality control of TGP also includes the determination of the contents of paeoniflorin, albiflorin, benzoylpaeoniflorin, and other related metabolites (Table 2). According to the Chinese Pharmacopoeia, the paeoniflorin content in *Paeonia lactiflora Pall.* botanical drugs should be standardized to not less than 1.6% (dry substance). Pu et al. used HPLC to determine the content of ethanol-water extracts of *Paeonia lactiflora Pall* (Pu et al., 2011). Furthermore, Ren et al. used ultra-high-performance liquid chromatography to determine the content of paeoniflorin in *Paeonia lactiflora Pall* (Ren et al., 2015).

**TABLE 2:** The pharmacologic study of TGP and its main active metabolites in dermatologic diseases.

Dermatologic diseases	Type of study	Drug	Experimental model	Mode of drug administration	Dose range tested	Duration	Results	Reference
Vitiligo	*In vivo*	PF	Monobenzone-induced vitiligo mice	i.p.	60 mg/kg	10 days	(+): Melanin content and intracellular tyrosinase activity of human melanocytes; protein levels of MITF and TRP-1; phosphorylation of CREB and ERK	Hu et al. (2020)
Vitiligo	*In vitro*	PF	H_2_O_2_-induced PIG1 and PIG3V	—	50 μM	24 h	(−): Cell apoptosis; suppressed ROS; H_2_O_2_-induced oxidative damage in PIG1 and PIG3V (+): Cell viability; the productions of SOD and CAT	Yuan et al. (2020)
Vitiligo	*In vitro*	PF	H_2_O_2_-induced PIG1 and PIG3V	—	50 μM	24 h	(−): The expressions of PDLIM1 and ROCK1 in PIG3V (+): The expressions of PDLIM1, RhoA and ROCK1 in PIG1; the expressions of RhoA and Nrf2 in PIG1, and ROCK1 and Nrf2 in PIG3V	Yuan et al. (2024)
Psoriasis	*In vivo*	PF	IMQ-induced psoriasis mice	i.p.	50, 100 mg/kg	7 days	(−): The infiltration of T cells, CD11c^+^DCs and neutrophils; the mRNA expression of IL-17, INF-γ, IL-6 and TNF-α	Chen et al. (2016)
Psoriasis	*In vivo*	PF	Psoriasis guinea pigs	i.p.	0.2 g per ear once a day	1 week	(−): IL-22 protein expression; the mRNA expression of IL-6, IL-17A and IL-22; protein expression of IL-22; p38 MAPK phosphorylation	Yu et al. (2017b)
Psoriasis	*In vivo*	TGP	Propranolol- induced psoriasis guinea pig; IMQ-induced psoriasis mice	i.g.	84, 168, 336 mg/kg; 180, 360, 720 mg/kg	2 weeks; 8 days	(−): Ear thickness; PCNA expression; psoriasis-like lesions; TNF-α, IFN-γ, IL-1β, IL-6, IL-12, IL-17, IL-22, IL-23 expression; TH-17 differentiation and keratinocytes proliferation; TH-1 recruitment in the epithelium; inflammatory response	Li et al. (2019a)
Psoriasis	*In vivo*	TGP	Psoriatic mice	—	50, 100, 200 mg/kg	14 days	(−): Skin pathological injury; epidermis layer thickness; the expression of toll-like receptor 4 and P65	Lei et al. (2023)
Psoriasis	*In vivo*	TGP	Psoriasis patients	oral	600–900 mg, 2 times/day	2 months	(−): The expression levels of STAT3 mRNA and PASI scores	Yang et al. (2015)
SS	*In vivo*	TGP	8-week-old female NOD mice	i.g.	100 mg/kg	16 weeks	(−): Anti-SSA/SSB; lymphocytic foci (+): Saliva flow rate; submandibular glands; ration of regulatory T cells	Li et al. (2013)
SS	*In vivo*	TGP	NOD mice	i.g.	1.2 mg/day	8 weeks	(−): The expression of ROR gammat mRNA (+): The expression of Foxp3 mRNA	Wu et al. (2021)
SS	*In vivo*	PF	SS mice	i.p.	150 mg/kg	42 days	(−): The expression of Cyr61; inflammation	Li et al. (2016a)
SS	*In vivo*	TGP	NOD mice	i.g.	400 mg/kg	4 weeks	(−): Overactivation of NLRP3 (Recombinant NLR Family, Pyrin Domain Containing Protein 3) inflammasome of submandibular gland cells.	Jiang et al. (2023)
SLE	*In vivo*	TGP	6–8 weeks old female MRL/lpr mice	i.g.	50 mg/kg	28 days	(−): The expression of ER alpha; Renal injury in SLE mice; Expression of IFN-gamma, IL-6, IL-12; dsDNA levels	Li and Jiang (2019)
SLE	*In vitro*	TGP	Human T cell	—	62.5, 312.5, 1,562.5 μg/mL	48 h	(+): Foxp3 expression; IFN- gamma and IL-2 expression	Zhao et al. (2012)
Urticaria	*In vivo*	PF	Urticaria-like mice	i.p.	100 mg/kg	10 days	(−): Scratching behavior and histopathological features; the serum levels of IgE, LTB4 and HIS; the number of mast cells and granules and diminished the infiltration of MCT and EPX; the inflammatory cytokine levels of IL-12 mRNA	Peng et al. (2022)
Urticaria	*In vivo*	PF	Rats with urticaria	oral	40 mg/kg	14 days	(−): Allergic and inflammatory; the production and release of IL-23; levels of P62 (+): Number of autophagosomes; levels of light chain 3B (LC3B) and Beclin-1; the expression of liver kinase B1 (LKB1) and AMP-activated protein kinase-α (AMPK-α); autophagic activity	Guo et al. (2021a)
OLP	*In vivo*	TGP	OLP patients	oral	0.6g/per time, 3 times/day	56 days	(+): IFN- gamma and IL-10 expression	Yan and Zhang (2016)
OLP	*In vitro*	TGP	Mesenchymal Stem Cell	—	0, 10, 100, 1,000 and 2000 μg/mL	6 h	(−):IL-6 and TNF-alpha expression; p-STAT3 expression (+): TGF-beta and IL-10 expression	Zhao et al. (2018b)
ACD	*In vivo*	TGP	2,4-Dinitrochlorobenzene -induced mice	i.g.	35, 70, 140 mg/kg/d	7 days	(−): Skin inflammation; the thymus and spleen indices; thymocyte proliferation; the production of IL-2 and IL-17 (+): IL-4 and IL-10 production	Wang et al. (2015)
ACD	*In vivo*	TGP	2,4-Dinitrochlorobenzene -induced mice	i.g.	200 mg/kg	14 days	(−): The levels of IgE and IL-6 in serum	Shen et al. (2021)
ACD	*In vivo*	TGP	ACD patients	oral	—	4 weeks	(−): The expression of proinflammatory cytokines.	Lei and Bo (2019)

Note: Abbreviations: SS, Sjogren’s syndrome; SLE, Systemic lupus erythematosus; OLP, Oral lichen planus; ACD, Allergic contact dermatitis; i. p., intraperitoneal injection; i. g., gastric lavage.

## 8 The pharmacological and clinical research progress of TGP and its main metabolites in the treatment of dermatologic diseases

Modern pharmacological studies have shown the diverse effects of TGP, such as anti-inflammatory, antioxidant, immunomodulatory, hepatoprotective, and analgesic, accompanied by mild adverse effects (Feng et al., 2019). Currently, *Paeonia lactiflora Pall.* is commonly utilized in managing rheumatoid arthritis (Lin et al., 2012; Wei et al., 2013), diabetic nephropathy (Xu et al., 2014; Shao et al., 2016), ankylosing spondylitis (Huang et al., 2019; Yang et al., 2023), hepatitis B (Zuo et al., 2017), obesity (Fang et al., 2023; Yao et al., 2024), and other diseases. In recent times, using TGP has been extended to the management of a wide range of dermatologic diseases encompassing vitiligo, psoriasis, contact dermatitis, lichen planus, sjogren’s syndrome and systemic lupus erythematosus. The diversity of pharmacological effects and low processing cost of peony make it ideal for treating dermatologic diseases (Gong et al., 2022) (Tables 2, 3). Despite the rich and well-established pharmacological effects of TGP, its utilization in dermatologic disease treatments is still in its infancy. Consequently, researchers globally are delving into the mechanisms underlying the action of TGP (Jiang et al., 2020).

**TABLE 3:** The clinical studies of TGP and its main active metabolites in dermatologic diseases.

Dermatologic diseases	Type of study	Drug/Treatment	Experimental model	Mode of drug administration	Dose range tested	Duration	Results	Reference
Vitiligo	*Clinical study*	TGP/NB-UVB	117 patients: treatment group (62), control group (55)	*oral*	Treatment group: TGP (0.6g/per time, 3 times/day) +UB-NVB (3 times/week); Control group: UB-NVB (3 times/week)	3 months	The efficacy of treatment group was better than that of the control group at 2 and 3 months	Gui et al. (2024)
Psoriasis	*Clinical study*	TGP/NB-UVB	60 patients: treatment group (30), control group (30)	*oral*	Treatment group: TGP (0.6g/per time, 3 times/day) +UB-NVB (3 times/week); Control group: UB-NVB (3 times/week)	8 weeks	The effective rate of treatment group:77.78%; The effective rate of control group:46.43%	Li et al. (2016b)
Psoriasis	*Clinical study*	TGP/Acitretin	108 patients: treatment group (54), control group (54)	*oral*	Treatment group: TGP (0.6g/per time, 2 times/day) + Acitretin (20 mg/d); Control group: Acitretin (20 mg/d)	12 weeks	The effective rate of treatment group:90%; The effective rate of control group:70.5%	Yu et al. (2017a)
SS	*Clinical study*	TGP/Shashen Maidongtang	84 patients: treatment group (42), control group (42)	*oral*	Treatment group: TGP (0.6g/per time, 2 times/day) +/Shashen Maidongtang (200 mL/per time, 2 times/day); Control group: TGP (0.6g/per time, 2 times/day)	3 months	The effective rate of treatment group:90.48%; The effective rate of control group:69.05%	Li et al. (2020b)
Urticaria	*Clinical study*	TGP/Levocetirizine/Montelukast	84 patients: treatment group (44), control group (40)	*oral*	Treatment group: Levocetirizine (5 mg/d)/Montelukast (10 mg/d)/TGP (0.6g/per time, 2 times/day); Control group: Levocetirizine (5 mg/d)/Montelukast (10 mg/d)	8 weeks	The effective rate of treatment group:86.36%; The effective rate of control group:50.00%	He et al. (2016)
SLE	*Clinical study*	TGP/Other treatments	Treatment group (792), control group (781)	—	—	—	Treatment group better than control group	Gong et al. (2022)
OLP	*Clinical study*	TGP/Wolfberry	70 patients: treatment group (35), control group (35)	*oral*	Treatment group: TGP (0.6g/per time, 3 times/day)/wolfberry 15 g/d; Control group: TGP (0.6g/per time, 3 times/day)	2 months	The effective rate of treatment group:88.57%; The effective rate of control group:68.57%	Li et al. (2016c)
OLP	*Clinical study*	TGP/0.1%Tacrolimus	38 patients: treatment group (20), control group (18)	*oral/ext.*	Treatment group: TGP (0.6g/per time, 3 times/day)/0.1%Tacrolimus (2 times/day); Control group: 0.1%Tacrolimus (2 times/day)	8 weeks	The effective rate of treatment group:75%; The effective rate of control group:44.4%	Yan and Zhang (2016)

Note: Abbreviations: SS, Sjogren’s syndrome; SLE, Systemic lupus erythematosus; OLP, Oral lichen planus; oral, profess conviction; ext., external.

### 8.1 Application of TGP in vitiligo

Vitiligo is a clinically common, limited pigment loss dermatologic disease that is not limited by age or location. The prevalence of vitiligo is about 0.5%–2% worldwide (Hu et al., 2023). The clinical features of vitiligo mainly include skin discoloration, showing well-defined ivory or chalky white patches. These patches can appear on the skin of various parts of the body, including the face, genitals, areola, and areas of repeated trauma, and can also involve mucous membranes (Ezzedine et al., 2015). Vitiligo can be classified into three types based on clinical characteristics: segmental vitiligo, non-segmental vitiligo, and mixed vitiligo (Du et al., 2023). Various external factors such as mental stress, trauma, and exposure to sunlight are closely related to the onset of vitiligo. The condition typically emerges when an individual, influenced by certain genetic factors, experiences the destruction of melanocytes due to various internal and external factors. This disruption impairs the production or melanization process ultimately leading to melanin loss. The mechanism of action of traditional Chinese medicine for vitiligo mainly involves tyrosinase, cholinesterase, melanocytes, immune function, cytokines, inflammatory mediators, and oxidative stress (Xie Y. H. et al., 2023).

Microphthalmia-associated transcription factors (MITF) is significantly involved in the regulation of melanogenesis and it can activate and regulate the expression of several key genes involved in melanin production, including tyrosinase and tyrosinase-related proteins (TRP-1 and TRP-2), thereby promoting melanin synthesis. Tyrosinase, recognized as the rate-limiting enzyme in melanogenesis, catalyzes tyrosine hydroxylation and oxidizes downstream DOPA to DOPAquinone for the spontaneous synthesis of melanin. In addition, TRP-1 and TRP-2 are involved in melanin biosynthesis downstream of tyrosinase (Liu et al., 2017; Pang et al., 2021; Di Bartolomeo et al., 2023). In a study by Hu et al., a mouse model of vitiligo was treated using PF at a level of 10 μg per milliliter to assess its impact and mechanism of action. The acquired data showed that the addition of PF significantly elevated melanin content and intracellular tyrosinase activity. This process upregulated the expression of MITF and TRP-1 through the ERK/CREB pathway (Hu et al., 2020). These processes positively affect the proliferation of human melanocytes, increase melanin biosynthesis, and attenuate the pathological changes in vitiligo mice. The resulting data affirmed the potential of PF for the treatment of vitiligo.

Oxidative stress plays a key role in the development and advancement of vitiligo. Imbalances in oxidative stress pathways such as decreased catalase levels, increased malondialdehyde, increased cell membrane lipid peroxidation, and dysregulation of superoxide dismutase have been observed in vitiligo patients. These events lead to elevated levels of overall oxidative/antioxidant balance in vitiligo patients, which manifests as increased levels of oxidative stress and decreased antioxidant capacity, ultimately leading to vitiligo onset and lesion progression (Jalel and Hamdaoui, 2009; Qiao et al., 2016). Notably, H_2_O_2_-induced oxidative stress is a key factor in the pathogenesis and progression of vitiligo. Yuan et al. demonstrated that PF activates the JNK/Nrf2/HO-1 signaling pathway and interacts with the PDLIM1/RhoA/ROCK1 pathway to protect SOD (PIG1, PIG3V) from oxidative damage. In addition, PF reduced the buildup of reactive oxygen species (ROS) and apoptosis by enhancing the levels of the antioxidant enzymes superoxide dismutase (SOD), catalase (CAT), MITF, and TRP-1. In addition, PF upregulated the expression of myoglobin, prostaglandin reductase 1 (PTGR1), LIM structural domain protein 1 (PDLIM1), Ras homologous gene family member A (RhoA), Rho-associated protein kinase (ROCK1), and F-actin in PIG1 cells. Conversely, in PIG3V cells, PF induced a downregulation of the expression of PTGR1, PDLIM1, ROCK1, and F-actin. Notably, these molecular changes were associated with an increase in melanin synthesis. The above studies demonstrated the potential therapeutic effects of PF in vitiligo, revealing that PF may inhibit oxidative stress through the Nrf2-ARE/HO-1 signaling pathway, thereby protecting melanocytes from oxidative damage (Yuan et al., 2020). In a separate investigation, Xiao found that PF promoted the proliferation of PIG3V cells. Subsequently, this process inhibited H_2_O_2_-induced expression of chemokine G protein-coupled receptor 17 (GPR17) and expression of IL-1β and IL-6 in PIG3V cells. These regulatory actions were achieved through the nuclear factor (NF)-κB pathway. Notably, this modulation of molecular pathways resulted in attenuating GPR17-mediated cytotoxic T-cell (CD8^+^T) migration to the site of inflammatory injury and reducing the inflammatory cascade of melanocyte response (Xiao, 2019).

The clinical treatment of vitiligo using TGP and its main active metabolites has also made certain research progress in addition to pharmacological studies. Gui et al. conducted a study to evaluate the efficacy of TGP combined with oral mini-pulse therapy (OMP) and narrow-band ultraviolet B (NB-UVB) in the treatment of active nonsegmental vitiligo (NSV). Data from 62 patients who received the TGP combination therapy and 55 patients who did not receive the TGP combination therapy were analyzed over a 3-month period. The results demonstrated that the combination of TGP with OMP and NB-UVB achieved superior therapeutic outcomes compared to OMP + NB-UVB alone. In the TGP group, the majority of patients experienced improvements in skin white patches during the course of treatment. Notably, there was no increase in side effects or recurrence rates in this group (Gui et al., 2024). Ye et al. utilized TGP combined with pimecrolimus ointment to treat patients with disseminated vitiligo. Clinical findings indicate that TGP may moderately elevate the CD4^+^/CD8^+^ T cell ratio and the level of CD4^+^CD25^+^ Treg cells in patients’ peripheral blood. These immunological adjustments are crucial for preserving the body’s immune balance. Therefore, TGP is implicated as one of the processes contributing to the healing of skin lesions in patients and in reducing the recurrence of skin lesions in vitiligo. However, this effect diminishes with increasing disease duration. In addition, TGP may provide a new option for patients with combined hepatic insufficiency due to its blood- and liver-nourishing effects (Ye et al., 2013).

In summary, TGP demonstrates positive effects on the clinical treatment of vitiligo and their adverse reactions are few. However, there are fewer current studies utilizing TGP in the treatment of vitiligo. This study presents a safe and promising new approach for treating refractory vitiligo clinically.

### 8.2 Application of TGP in psoriasis

Psoriasis, a chronic inflammatory dermatologic disease, is marked by abnormal T-cell activation, infiltration, and excessive growth of skin keratinocytes (Kim et al., 2017). The disease’s development is associated with immune inflammation, oxidative stress, and imbalances in cell growth and apoptosis (Chimenti et al., 2018). Currently, traditional retinoids, vitamin D3 derivatives, glucocorticoids, immunosuppressants, and biologics are still the mainstay of treatment for psoriasis (Zhang L. H. et al., 2024). Despite being the primary approach, these treatments come with limitations, including significant adverse effects, high relapse rate, long duration of disease, and high price. Hence, the exploration of new methods that offer a high cure rate, are cost-effective, and exhibit lower adverse reactions is extremely important. In recent years, traditional Chinese medicine’s active metabolites have become prominent in psoriasis treatment by virtue of their high cure rate, affordability, and low incidence of adverse effects (Wang and Liu, 2004; Guo et al., 2024; He et al., 2024). TGP has emerged as one such metabolite, exhibiting promising research outcomes and gaining widespread utilization in the treatment of psoriasis.

The main pathological changes in psoriasis include excessive keratinocyte proliferation, epidermal hyperplasia, and increased neoangiogenesis, accompanied by inflammatory cell infiltration (Luo et al., 2020; Orsmond et al., 2021; Pandey et al., 2021). Vascular endothelial growth factor (VEGF), a strong promoter of angiogenesis, is rarely expressed or even absent in normal epidermal cells. However, it is abundantly expressed in keratinocytes of patients with psoriasis, especially in lesions. Therefore, VEGF has the potential to produce psoriasis-like lesions and typical Koebner-like psoriasis damage by inducing a vascular inflammatory response (Voiculescu et al., 2014; Chen et al., 2023). Yu’s study showed that PF had an inhibitory effect on keratinocyte growth factor (KGF)-induced HaCaT cell proliferation. This effect is thought to be achieved by PF through the regulation of the p38 MAPK/NF-κB p65 signaling pathway, which downregulates IL-22 and VEGF mRNA expression (Yu et al., 2007). Zhang’s study found that TGP reduced VEGF mRNA expression in the skin of psoriatic mice. Therefore, TGP may treat psoriasis by inhibiting keratinocyte proliferation and neoangiogenesis (Zhang, 2015).

The onset and progression of psoriasis are closely related to immune dysfunction. In addition to keratinocytes in the skin, immune cells, and their cytokines are crucial in the pathogenesis of psoriasis (Zhao X. T. et al., 2018; Shin et al., 2020). Dendritic cells (DCs) and macrophages in the dermis of psoriasis produce IL-23, which induces the activation of immune cells such as Th1 cells and the release of inflammatory cytokines like IL-22, IL-6, and tumor necrosis factor-α (TNF-α). These inflammatory cytokines act on keratin-forming cells, leading to typical pathological changes associated with psoriasis such as hyperkeratosis of the epidermis and hypertrophy of the stratum spinosum (Zhao et al., 2022). Paeoniflorin, the main metabolite of TGP, can inhibit the differentiation, maturation, and function of DCs, which provides a theoretical basis for the treatment of autoimmune diseases with *Paeonia lactiflora Pall* (Zhou et al., 2012; Sun et al., 2015). Li et al. found that TGP could reduce psoriasis-like damage, such as erythema, scaling, and inflammatory cell infiltration of the auricular skin of mice induced by imiquimod. Furthermore, TGP exhibited the potential to decrease the proliferation of keratin-forming cells. Mechanistically, TGP exerts antiproliferative effects by blocking the phosphorylation of total or phosphorylated signal transducers and activators of transcription (STAT1 and STAT3) in skin lesions, inhibiting their mRNA expression and T-helper 17 (Th17) cell differentiation, and downregulating IL-17A and IL-22 levels (Li B. B. et al., 2019). According to Zhao’s study, PF inhibited imiquimod-induced psoriasis. They noted that PF reduced keratinocyte proliferation and inflammatory cell infiltration, and decreased Th17 cytokine mRNA expression. In addition, PF did not affect splenocyte viability but decreased IL-17 secretion under Th17-polarized conditions. PF inhibited Th17 cytokine mRNA expression and STAT3 phosphorylation in splenocytes under Th17-polarized conditions. These findings suggest that PF can inhibit imiquimod-induced psoriasis by modulating Th17 cell responses and cytokine secretion through STAT3 phosphorylation (Zhao et al., 2016).

As a chronic inflammatory dermatologic disease, psoriasis is closely linked to chronic inflammatory responses mediated by T cells and epidermal cells (Reich et al., 2001; Homey and Bunemann, 2004; Liao et al., 2023). Reducing the level of pro-inflammatory cytokines can attenuate the inflammatory response at the skin lesions of psoriasis mice, which in turn improves the symptoms of skin lesions (Wang et al., 2023). Sun et al. established a mouse model of psoriasis. In addition, they observed that PF improved psoriasis lesions and decreased the number of F4/80^+^CD68^+^ macrophages, CD11b^+^Gr-1^+^ neutrophils, and their secretion of cytokines (TNF-α, IL-1β, IL-6, IL-12, and IL-23), inducible nitric oxide synthase (iNOS), and macrophage inflammatory protein-2 (MIP-2) in the lesions. In addition, PF downregulated the expression of Th1/Th17-related cytokines (Sun et al., 2015). In another study, Yu et al. found that TGP-treated psoriasis mice exhibited a reduction in IL-22 and p38 proteins, as well as a decrease in the mRNA levels of IL-6, IL-17A, and IL-22. Therefore, TGP can inhibit the phosphorylation of the p38 MAPK pathway, downregulate the expression of VEGF, IL-23, and its proteins in HaCaT cells, and reduce the expression levels of IL-6, IL-17A, IL-22, IL-23, thus exerting anti-inflammatory and antiproliferative effects (Yu et al., 2007).

Research indicated that the application of TGP in conjunction with other drugs or physiotherapy was effective in treating psoriasis. In addition, this treatment can reduce adverse effects in individuals and improve their adherence to the treatment (Tang et al., 2014). Currently, psoriasis is usually treated with TGP in combination with retinoids or narrow-spectrum medium-wave ultraviolet light (Li T. T. et al., 2019). In a search for alternative treatments, Yu et al. found that the combination of TGP and acitretin not only improved the therapeutic efficacy of psoriasis, but also reduced the liver damage caused by acitretin (Yu C. et al., 2017). A multicenter, double-blind, placebo-controlled, randomized clinical trial on the efficacy and safety of acitretin combined with TGP in the treatment of moderate-to-severe plaque psoriasis (108 cases) was conducted. The resulting data showed that the body surface area (BSA) and psoriasis area and severity index (PASI) values of the experimental group (20–30 mg/d of acitretin +1.8 g/d of TGP for 12 consecutive weeks) were markedly reduced in contrast to the control group (20–30 mg/day of acitretin + placebo for 12 consecutive weeks). In addition, the serum aminotransferase levels of the former group were lower than those of the latter group, suggesting that the combination of TGP treatment can increase the clinical efficacy and reduce the hepatotoxicity of acitretin (Yu et al., 2015). Thus, this implies that the combination therapy not only decreased PASI scores, but also reduced hepatotoxicity of chemical drugs and disease recurrence.

In conclusion, TGP can treat psoriasis by inhibiting keratinocyte hyperproliferation and neoangiogenesis, as well as by immunomodulation and anti-inflammatory responses. However, its specific mechanism of action still needs further study. TGP can be used alone as a therapeutic drug for psoriasis, or combined with other drugs and physical therapy to treat psoriasis. These findings have important reference value for the future clinical treatment of psoriasis.

### 8.3 Application of TGP in Sjogren’s syndrome

Sjogren’s syndrome (SS) is a chronic inflammatory autoimmune disease characterized by lymphocyte infiltration of exocrine glands (Alexopoulou, 2022). Clinical manifestations of the syndrome include dryness of the mouth and eyes and the presence of autoantibodies and hyperimmunoglobulins in the serum of the patient (Ren et al., 2023; Zhang A. P. et al., 2023). However, the exact etiology and pathogenesis of desiccation syndrome remain to be further elucidated.

Animal model studies have found that TGP can reduce the concentration of cytokines and autoantigens in the serum of experimental mice, and attenuate the inflammatory response in SS mice (Li B. B. et al., 2020). Using a non-obese diabetic mouse model (NOD), Li et al. found that salivary gland flow rate and the ratio of splenic regulatory T-cells were elevated in the PF-treated group of mice in contrast to the saline control group. However, serum anti-SSA/SSB levels, and lymphocytes were significantly reduced. Furthermore, SS treatment with PF was found to increase the expression of aquaporin (AQP)-5, alleviate inflammation, and restore autoantibody levels in the NOD mouse model. Therefore, PF can restore the function of salivary glands and delay the appearance of early clinical symptoms of Sjogren syndrome (Li et al., 2013).

Cysteine-rich (Cyr61) has been gradually acknowledged as a new inflammatory factor involved in diverse autoimmune and inflammatory diseases (Kular et al., 2011). Li et al. found that Cyr61 expression was upregulated in the salivary glands of SS patients, and also in the submandibular glands of experimental mice. In addition, inhibition of Cyr61 expression in experimental mice alleviated the production of inflammatory cytokines and improved salivary gland secretion. These results suggested that Cyr61 is involved in the progression of SS. PF treatment significantly downregulated the expression of Cyr61 and alleviated the symptoms of SS mice, indicating that PF alleviates SS by inhibiting the expression of Cyr61 (Li H. et al., 2016).

Regarding relevant clinical research, a clinical trial showed that TGP (1.8/d × 24 w) significantly improved dry mouth and eyes, and reduced disease activity and erythrocyte sedimentation rate (ESR) levels in primary SS patients. Additionally, the treatment was well tolerated with mild adverse effects (Liu et al., 2019). Another meta-analysis of the application of TGP in the treatment of SS also showed that TGP significantly improved the function of lacrimal secretion, and its efficacy was better than that of the placebo group. In addition, TGP combined with immunosuppressive drugs (e.g., hydroxychloroquine) not only promotes gland secretion and relieves the dry mouth and eyes of patients, but also reduces inflammatory indices (e.g., ESR, serum C-reactive protein (CPR)) and plasma immunoglobulin (e.g., gamma globulin, IgG, IgA, and IgM) levels. In addition, this combination therapy was safe and well tolerated, demonstrating the advantages of combining TGP in the treatment of Sjogren syndrome (Feng et al., 2019).

In summary, TGP exhibited good therapeutic effects on SS and is free of hepatotoxicity and ocular toxicity. However, the onset of action of TGP is slow, indicating that therapeutic effects might take some time to become evident. Therefore, the development and application of TGP still need to be further explored.

### 8.4 Application of TGP in lupus erythematosus

Lupus erythematosus is a chronic autoimmune disease in which the production of autoantibodies causes damage to tissues and organs of the body, resulting in severe systemic damage, i.e., systemic lupus erythematosus (SLE) (Guo X. R. et al., 2021; Qiu et al., 2022; Viatchenko-Karpinski et al., 2023). The pathogenesis of SLE is complex, with the involvement of a multitude of factors in different genetic backgrounds. In SLE, the number and function of T lymphocytes, B lymphocytes, and many cytokines (IL-1, IL-10, TNF-α) are significantly abnormal, and there is obvious heterogeneity in the pathogenesis (Perl, 2012). SLE is characterized by an abnormality in multiple immunomodulatory functions. Therefore, immunomodulation becomes an important tool in the clinical treatment of SLE. Currently, the conventional treatment of SLE mainly involves glucocorticoids and immunosuppressants (Ugarte-Gil and Alarcón, 2014; Zhang L. C. et al., 2024). However, the adverse effects of these drugs limit their clinical use.

Animal experiments on the early application of TGP in SLE showed that TGP exhibited a bidirectional regulatory effect (promoting effect at low concentrations and inhibitory effect at high concentrations) on the proliferative response of splenic lymphocytes induced by sabinin A in mice. In addition, TGP can promote phagocytosis of peritoneal macrophages and partially or completely antagonize the increase of serum IgG-type autoantibody levels in mice, which is protective against SLE-like changes in mice (Zhang and Wei, 2020). In the active phase of SLE, the decrease of Treg cells in the blood correlates with the severity of the disease. Zhao et al. gave different concentrations of TGP to Lupus patients and controls (healthy individuals). The resulting data indicated a significant increase in CD4^+^CD25^+^ Treg cells in Lupus patients, whereas no significant change was observed in the controls (Zhao et al., 2012). Forkhead/winged helix transcription factor (Foxp3), the forkhead gene for transcription factors, primarily regulates Treg cell progression (Fontenot et al., 2003). Expression of the Foxp3 gene induces the conversion of CD4^+^CD25^−^ T cells to CD4^+^CD25^+^ Treg cells (Hori et al., 2003). In addition, the use of TGP resulted in upregulating the expression level of Foxp3, causing Foxp3 demethylation and increasing the production of IFN-γ and IL-2, thereby promoting the expression of Treg cells in CD4^+^ T cells. Another study showed that the expression level of CD4^+^CD25^+^ T cells in patients with active SLE was notably lower than that in healthy controls. In addition, TGP combined with immunosuppressive agents (e.g., tacrolimus) significantly increased CD4^+^CD25^+^ T-cell expression in patients, decreased DAI, increased clinical efficacy, and reduced the incidence of disease recurrence (Li et al., 2018).

From clinical research perspective, Zhang et al. conducted a clinical study on the therapeutic effects of combining glucocorticoids, mycophenolate mofetil, and total paeoniflorin capsules in the treatment of SLE. They selected 84 patients with SLE and randomly divided them into a control group and a treatment group, with 42 patients in each group. The control group was treated with prednisone and mycophenolate mofetil, while the treatment group received additional TGP capsules based on the control group’s regimen and the treatment lasted for 6 months. The study results showed that the combination of glucocorticoids, mycophenolate mofetil, and TGP capsules was more effective in treating SLE than the combination of glucocorticoids and mycophenolate mofetil alone (Zhang Y. et al., 2023). Chen et al. conducted a meta-analysis on the treatment of SLE with TGP to assess the efficacy of TGP. The study included data from 14 RCTs with 978 patients (492 in the trial group and 486 in the control group). Their results showed a significant improvement in the SLE disease activity index (DAI), an elevation in serum complement levels, and a decrease in blood sedimentation in the test group (TGP combined with glucocorticoids or cyclophosphamide) in contrast to the control group (not combined with TGP). In addition, the average daily dose of glucocorticoid and the cumulative dose of cyclophosphamide were reduced in the experimental group, and the rate of disease recurrence and adverse effects were also reduced. The acquired data demonstrate the effective role of TGP in treating SLE (Chen et al., 2022).

In conclusion, TGP, either alone or in combination, is effective in the treatment of SLE. In addition, TGP exhibited a good hepatoprotective effect, and the adverse effects were only slight gastrointestinal reactions (Wang et al., 2022). These findings provide new ideas for the clinical treatment of SLE.

### 8.5 Application of TGP in urticaria

Urticaria is a limited edematous reaction, characterized by wheals and erythema, caused by dilation and increased permeability of small blood vessels in the skin and mucous membranes (Deng et al., 2020). Chronic urticaria (CU) occurs in about 0.1% of the general population and is increasing (Xiao et al., 2023). CU is categorized into two main types: spontaneous and induced (Bartko et al., 2023). The etiology of the former is complex and involves both immune and non-immune pathogenesis. Of these, type I hypersensitivity is the most common mechanism of reaction. Immunoglobulin IgE mediates mast cell degranulation, releasing a variety of inflammatory chemical mediators, mainly histamine, to induce urticaria (Bracken et al., 2019). The clinical management of CU is complex. Despite the effectiveness of histamines, recurrence may occur after discontinuation (Claveau et al., 1993). In addition, immunosuppressants (e.g., cyclosporine A, Tripterygium wilfordii) are effective in only some patients and have significant toxic side effects (Khan, 2013; Kitsioulis et al., 2017). These limitations greatly restrict the use of these drugs. In recent years, TGP has been applied to the treatment of urticaria.

Peng et al. (2022) applied PF to an animal model of urticaria. The experimental results showed that PF significantly alleviated scratching behavior and histopathological features. In addition, PF reduced the cellular levels of immunoglobulin E (IgE), leukotriene B4 (LTB4), and histamine (HIS) in serum, markedly reduced the number of mast cells and granules in the skin tissues of urticaria mice, and decreased the infiltration of inflammatory cells (MCT and EPX). Oral administration of PF was effective in reducing inflammatory cytokine IL-12 mRNA levels. Another animal model study exhibited that PF inhibited allergic and inflammatory responses and ameliorated urticarial lesions by mitigating pathologic abnormalities, mast cell infiltration, and attenuating histamine secretion. Mechanistically, the application of PF significantly limited the production and release of the inflammatory cytokine interleukin (IL-23), whereas levels of IL-17 remained unchanged. In addition, PF intervention increased the number of autophagosomes, suggesting that PF enhances autophagic activity in urticarial lesions. Additionally, PF treatment elevated the expression of liver kinase B1 (LKB1) and AMP-activated protein kinase α (AMPKα), which promoted PF-enhanced autophagic activity. Thus, PF inhibits the inflammatory cytokine IL-23 via the LKB1/AMPK-α pathway, enhances autophagic activity, and effectively ameliorates urticarial lesions (Guo J. et al., 2021).

In the area of clinical research, He et al. investigated the efficacy and safety of combining levocetirizine dihydrochloride with montelukast sodium and total glucosides of paeony in the treatment of chronic spontaneous urticaria. A total of 84 patients were randomly assigned to two groups: the treatment group (44 patients) and the control group (40 patients). The treatment group received oral levocetirizine dihydrochloride 5 mg once daily, montelukast sodium 10 mg once daily, and total glucosides of paeony 0.6 g twice daily. The control group received only oral levocetirizine dihydrochloride 5 mg once daily and montelukast sodium 10 mg once daily. Both groups underwent an 8-week treatment course, with disease severity scores recorded before treatment and at weeks 2, 4, 6, and 8 during treatment. Clinical efficacy and adverse reactions were assessed at the end of the treatment period. The results showed that after 8 weeks, the reduction in disease severity scores was greater in the treatment group than in the control group, with an efficacy rate of 86.36% in the treatment group compared to 50.00% in the control group (He et al., 2016). Another clinical observational study evaluating the combination of TGP with cetirizine in the treatment of urticaria exhibited favorable results within the treatment group (Long et al., 2010). That study showed that during the initial two-week dosing period, the treatment group with the combination of TGP and cetirizine showed significantly better effects on symptom improvement than the control group with cetirizine alone. The serum IL-4 and IgE levels were significantly lower in the treatment group, and the 1-month relapse rate was also low. Therefore, TGP exhibited the potential to improve the condition of CU patients and reduce the relapse rate by regulating the immune function.

In summary, TGP as a supplementary treatment can provide positive outcomes for CU in adolescents and adults, with minimal and manageable side effects (Li et al., 2022). However, future studies require more high-quality, long-term clinical trials to provide more robust evidence for clinical practice.

### 8.6 Application of TGP in oral lichen planus

Oral lichen planus (OLP) is a non-infectious superficial inflammatory disease of the oral mucosa (Didona and Hertl, 2022). The recurrent and cancerous potential of OLP is detrimental to the quality of life and psychological wellbeing of the afflicted individuals. The pathology of OLP is characterized by a dense subepithelial lymphocytic infiltrate, destruction of the basement membrane, and apoptosis of keratinocytes (Deng et al., 2022; Geng et al., 2022; Qing et al., 2023). Currently, the etiology of OLP is unclear and requires further elucidation, although research has implied the involvement of a variety of factors in this disease. The pathogenesis of OLP is closely related to immune factors, and it is a chronic disease characterized by T-lymphocyte-mediated immune response (Liu et al., 2014). Antigen-specific and non-specific mechanisms may be involved in the immune process of OLP. Specifically, the uptake and processing of unknown antigens by DCs triggers an immune response. This response results in the accumulation of T lymphocytes and other immune cells at the lesion site and the secretion of a large number of cytokines. Subsequently, these events contribute to the degradation of the basement membrane and the destruction of keratinocytes, resulting in inflammatory lesions of the oral mucosa (Gobbo et al., 2017; Park et al., 2018).

Wang et al. found high expression of Toll-like receptor 4 (TLR4) and activation of the NF-κB signaling pathway in tissues of patients with oral lichen planus. In addition, keratinocytes from OLP patients showed higher levels of mRNA expression of inflammatory factors interleukin-6 (IL-6) and TNF-α than normal oral epithelial keratinocytes. The local inflammation model of OLP constructed by lipopolysaccharide (LPS)-stimulated keratinocyte HaCaT cells was assessed concerning the impact of TGP application. The data indicated that TGP could dose-dependently suppress the LPS-induced expression of IL-6 and TNF-α in these cells. In addition, TGP treatment reduced the phosphorylation of NF-κB inhibitory protein α and NF-κB p65 protein, causing a reduction in NF-κB p65 nuclear transcription in HaCaT cells (Wang et al., 2016). A clinical observation of TGP combined with glucocorticoids in the treatment of OLP showed that TGP is a safe and effective drug with few side effects in the treatment of OLP. In addition, TGP combined with glucocorticoids exhibits a definite therapeutic effect (Zhou L. L. et al., 2016).

Mesenchymal stem cells (MSCs) have been employed in both clinical and experimental settings to mitigate severe immune-linked diseases utilizing their immunomodulatory properties. However, these beneficial properties are impaired in the presence of inflammation (Park et al., 2018). Zhang et al. explored the immunomodulatory effects of PF on MSCs and the potential role of Th1/Th2 cytokines in OLP. They observed that PF promoted the proliferation, migration, and multilineage differentiation of MSCs in OLP lesions by regulating the Th1/Th2 balance. This resulted in prolonging graft survival time and improving inflammatory infiltration. It was observed that PF enhances MSC immunomodulation and regulates the inflammatory microenvironment via T lymphocytes. Furthermore, it offers a promising therapeutic measure for OLP treatment by improving MSC function (Zhang et al., 2022b). In another study, MSCs from OLP showed elevated levels of IL-6, TNF-α, transforming growth factor β (TGF-β), and IL-10 in comparison to the control group. TGP significantly improved the immunomodulatory function of MSCs via the inhibition of IL-6 and TNF-α expression and elevation of the expression of TGF-β and IL-10. In addition, TGP could inhibit the expression of p-STAT3 by upregulating miR-124. Alterations in IL-6, TGF-β, and p-STAT3 were mediated by overexpression and knockdown of miR-124 in MSCs. Thus, TGP may significantly decrease pro-inflammatory cytokines and increase anti-inflammatory mediators through the miR-124/STAT3 pathway (Zhao Z. F. et al., 2018).

From a clinical research perspective, Wu et al. studied the short-term effects of combined treatment with compound phellodendron gargle and TGP capsules on OLP. Sixty-two patients were randomly divided into a control group and an observation group. Both groups received oral TGP capsules, while the observation group additionally used compound phellodendron gargle as a mouth rinse. The treatment effects were evaluated after 30 days based on the Visual Analog Scale (VAS) for pain and clinical signs assessment. The results showed significant improvement in the VAS and clinical signs scores in the observation group, as well as an improvement in the VAS scores in the control group, compared to pre-treatment levels (Wu and Ma, 2023).

### 8.7 Application of TGP in allergic contact dermatitis

Allergic contact dermatitis (ACD) is a delayed-type hypersensitivity reaction (type IV hypersensitivity reaction) mediated by semi-antigen-specific T cells, which is produced by T cells after allergen contact with the skin and is stimulated by antigen-presenting cells (Scheau et al., 2020). It occurs in two phases: the sensitization and stimulation phases (Weidinger and Novak, 2016). Notably, a small portion of ACD may also present as the delayed phase of type I hypersensitivity reaction. Allergic contact dermatitis is mainly characterized by a series of skin inflammatory cell infiltration and the release of inflammatory mediators in the local skin after antigenic stimulation (Zhou P. et al., 2016). The main clinical manifestations of allergic contact dermatitis are redness, edema, and itching. Repeated exposure to allergens at the lesion site can lead to recurrent episodes of ACD and even trigger atopic dermatitis and disability, seriously affecting the quality of life of the patients (Wong et al., 2015). ACD affects a wide range of populations, with a global prevalence rate of up to 15%–20%, across all age groups. Specifically, ACD exhibits a prevalence of 15.2% in adolescents and 18.6% in adults (Aloui et al., 2022). Exposure to the work environment, age, gender, dietary habits, and genetic predisposition are considered to be the most important risk factors. Currently, western medical treatment of ACD mainly involves the use of topical corticosteroids and antihistamines, which are highly effective (Ta et al., 2021). However, these drugs may trigger serious adverse reactions such as hypercorticism, muscle atrophy of hands and feet, liver function damage, central inhibition, and cardiotoxicity. In recent years, researchers have applied TGP for the treatment of ACD, yielding promising research results.

Dendritic cells (DC) are important antigen-presenting cells, and there are at least two subpopulations of DCs in steady state: intraepidermal Langerhans cells and intradermal DCs (Ouchi et al., 2016). DCs carrying allergens migrate to local draining lymph nodes, where they selectively induce the activation of antigen-specific T cells through the presentation of MHC-II molecules. This results in initiating the development of a type IV allergic response. In addition, DCs also produce various cytokines that regulate the differentiation of T lymphocytes. Therefore, inhibiting the migration of LCs/DCs and the activation of T cells is considered to be an important method to control ACD (Kimber et al., 2012). Using an ACD mouse model, Shi et al. observed that PF increased the expression of a cytokine signaling suppressor (Socs3), which is highly expressed by DCs at both the protein and gene levels. In addition, high expression of Socs3 inhibited IL-6/STAT3 signaling and activation of downstream signaling pathways, thereby suppressing IL-6 levels and TH17 cell proliferation (Shi et al., 2016).

In addition, T-lymphocytes are the main effector cells in the disease progression of ACD. Activation of T lymphocytes by antigen-presenting cells results in the production of a large number of cytokines: IL-2, IL-4, and IL-1. Among these, an imbalance of IL-2 and IL-4 cytokines promotes the progression of hypersensitivity (Popov et al., 2011). Overproduction of IL-17 and underproduction of IL-10 have also been found in ACD patients. It was noted that IL-17 exacerbates the degree of hypersensitivity and increases the production of inflammatory cytokines. However, IL-10 inhibits antigen-presenting cells to relieve the inflammatory response and even improves immune tolerance, reducing IL-17 levels (Harrington et al., 2005).

Wang et al. observed that PF significantly alleviated the degree of ear swelling and infiltration of inflammatory cells and inhibited the proliferation of thymocytes through the establishment of a mouse model of DNCB-induced ACD (Wang et al., 2013). Assessment of the cytokines in serum, thymus, and spleen supernatants revealed elevated levels of IL-4 and IL-10, and decreased levels of IL-2 and IL-17. These findings indicate that PF has a regulatory function in ACD, regulating the balance between inflammatory and anti-inflammatory cytokines. In addition, PF inhibited the production of the inflammatory stimulating factor IL-12 and increased the expression of IL-10 and TGF-β. This may be another mechanism by which PF modulates DC-induced T-cell immune tolerance for the effective treatment of inflammatory and immune-related diseases such as allergic contact dermatitis. It is well known that cytokines IL-2, IL-4, IL-10, and IL-17 represent Th1, Th2, Treg, and Th17 cells, respectively. The differentiation of T lymphocytes is primarily governed by the signaling pathway JAK-STAT transcription factors (Jin et al., 2012). In addition, the expression of aberrant transcription factors (T-box family of novel transcription factors, zinc finger transcription factors, forkhead family of transcription factors, and tretinoin-associated orphan nuclear receptor) causes an imbalance in the ACD cytokine network (Yin et al., 2011). Therefore, PF has the potential to regulate the janus kinase/signal transducers and activators of transcription (JAK/STAT) transcription factor pathway to correct the aberrant differentiation of T cells and ultimately restore the balance of the cytokine network (Gangwar et al., 2021). However, this hypothesis needs to be further verified. Shi et al. established a mouse model of allergic contact dermatitis, which further demonstrated that PF could inhibit the ability of DC to stimulate the proliferation of allogeneic T cells. Furthermore, PF activated the differentiation of primitive T cells into CD4^+^ T cells to produce interferon-γ, which induced the production of IL-10 by CD4^+^CD25^+^Foxp3^+^ T cells, thus alleviating the inflammatory response (Shi et al., 2014). In summary, PF exhibited effectiveness in preventing and treating ACD inflammation *in vitro* by inhibiting the maturation of DCs and limiting their ability to activate T lymphocytes.

In the context of clinical research on ACD, Shu et al. investigated the efficacy of TGP in treating children with atopic dermatitis and monitored for adverse reactions. They selected 40 pediatric patients with ACD and randomly divided them into two groups. The control group, consisting of 20 patients, was treated with oral cetirizine and topical mometasone furoate ointment. The observation group, also consisting of 20 patients, received oral TGP in addition to topical mometasone furoate ointment. The results indicated that TGP significantly improved the treatment outcomes for children with atopic dermatitis, with no significant adverse reactions observed (Shu et al., 2021).

### 8.8 Application of TGP in other dermatologic diseases

TGP has also been applied in treating other dermatologic diseases like alopecia areata (Yang et al., 2012; Yang et al., 2013), alopecia (Zhang T. et al., 2021; Drake et al., 2023), hyperpigmented dermatologic diseases (Qiu et al., 2016), radioactive skin damage (Kong et al., 2016; Lu et al., 2020), bromhidrosis (Xu et al., 2022), and scleroderma (Li et al., 2023) owing to its anti-inflammatory, antioxidant, and immune-regulating properties. However, the utilization of TGP for the treatment of these dermatologic conditions is still a small-sample exploratory study or a case study treatment experience. In addition, the intensity of evidence-based medicine is limited. Therefore, future in-depth large-sample, multicenter clinical trial studies are needed for further validation.

## 9 Mechanisms of action and targeting signaling pathways of TGP and its main metabolites in dermatologic diseases

In recent years, TGP has garnered increasing attention in the treatment of dermatologic diseases.

**FIGURE 2:**
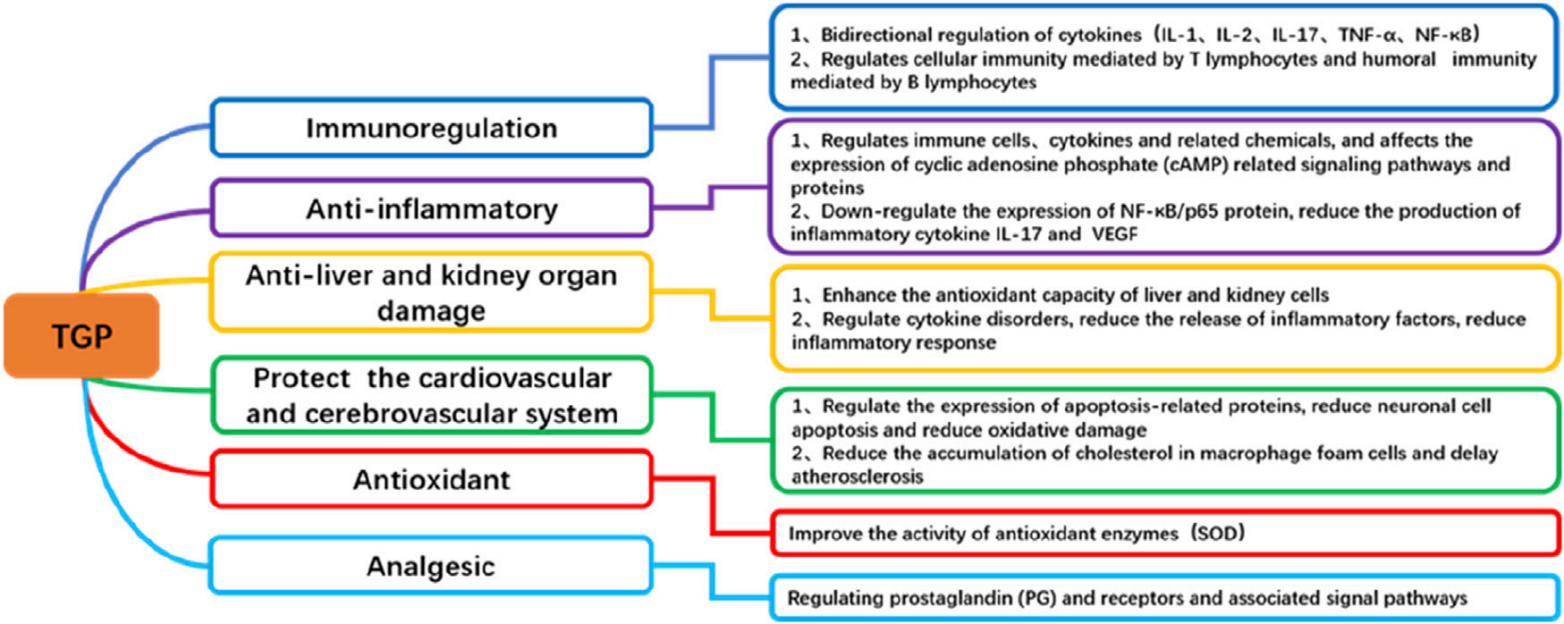
Pharmacological effects of TGP.

**FIGURE 3:**
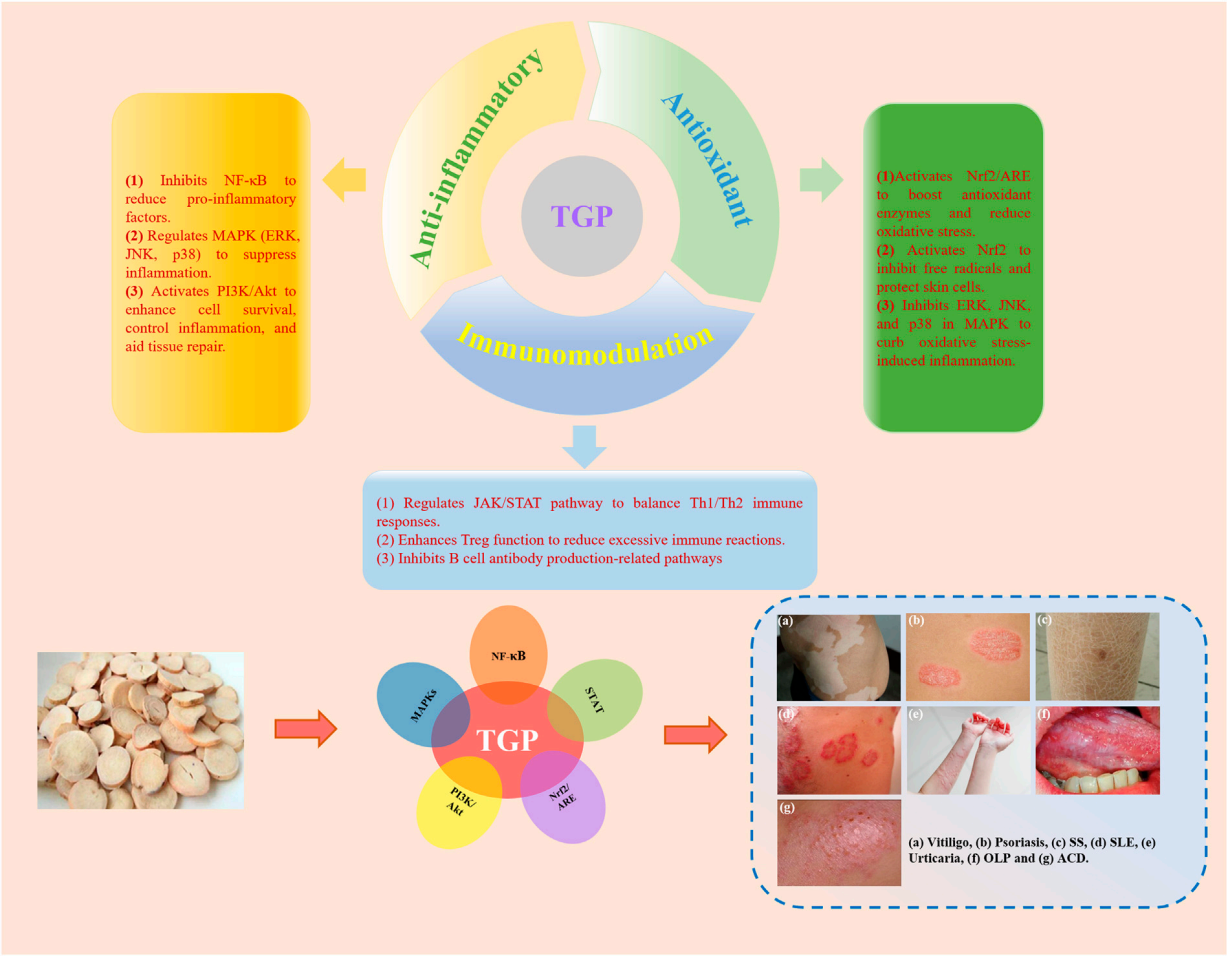
The main targeted signaling pathways and action mechanism of TGP in the treatment of dermatologic diseases.

TGP exhibits a wide range of pharmacological effects (Figure 2), but its mechanism of action in treating dermatologic diseases primarily relies on its anti-inflammatory, antioxidant, and immunomodulatory functions (Türsen et al., 2020). By regulating multiple cellular signaling pathways, including NF-κB, STAT, MAPKs, PI3K/Akt, Nrf2/ARE and others, TGP can effectively alleviate skin inflammation, modulate immune responses, and suppress oxidative stress, thereby achieving therapeutic effects in dermatologic diseases (Figure 3).

### 9.1 Anti-inflammatory

One of the key pharmacological properties of TGP in the treatment of dermatologic diseases is its potent anti-inflammatory effect. Inflammatory dermatologic diseases, such as psoriasis, urticaria, atopic dermatitis, and others, are often characterized by pronounced inflammatory responses, which TGP effectively mitigates through multiple mechanisms (Zhang Y. et al., 2021). TGP inhibits the activation of NF-κB, thereby reducing the release of pro-inflammatory cytokines and alleviating inflammation. For instance, in OLP, TGP has been shown to suppress the production of inflammatory cytokines by inhibiting the NF-κB signaling pathway (Wang et al., 2016). Additionally, TGP exerts its anti-inflammatory effects by inhibiting key molecules in the MAPKs signaling pathway, including ERK, JNK, and p38, which are critical in the cellular inflammatory response. By modulating the MAPKs pathway, TGP decreases the expression of pro-inflammatory factors, reduces cytokine release, and alleviates skin inflammation and tissue damage (Dawid-Pac, 2013). In chronic inflammatory dermatologic diseases, such as ACD, psoriasis. TGP exerts its anti-inflammatory effects by regulating the STAT pathway, further reducing inflammation (Yang et al., 2015; Shi et al., 2016). Moreover, TGP decreases the release of various inflammatory mediators, such as prostaglandins, leukotrienes, and histamine, which are key contributors to the skin’s inflammatory response. By inhibiting their production, TGP effectively alleviates inflammatory reactions in both acute and chronic dermatologic diseases (Hongying et al., 2011).

Additional research has indicated that TGP plays a significant role through the regulation of the PI3K/Akt signaling pathway. The PI3K/Akt pathway is critical for cell growth, proliferation, differentiation, and survival, with particular importance in the regulation of inflammatory responses and tissue repair. By activating this pathway, TGP promotes the survival of skin cells, inhibits excessive inflammatory reactions, and accelerates tissue repair (Zhao et al., 2022).

### 9.2 Antioxidant

TGP plays a crucial antioxidant role in the treatment of vitiligo, ACD, and psoriasis by modulating oxidative stress responses and associated signaling pathways, effectively reducing inflammation and mitigating tissue damage (Kong et al., 2016). In vitiligo, TGP lowers the generation of ROS, thus protecting melanocytes from oxidative stress-induced injury. This protective effect is mediated through the activation of the Nrf2 signaling pathway, which upregulates antioxidant enzymes such as SOD, CAT, and glutathione peroxidase (GSH-Px), promoting the elimination of ROS and reducing cellular damage (Yuan et al., 2024). Additionally, TGP alleviates oxidative stress-induced damage to melanocytes by modulating the MAPKs signaling pathway. Furthermore, TGP inhibits the activation of the NF-κB signaling pathway, reducing the production of pro-inflammatory factors and thereby attenuating skin inflammation. By suppressing inflammation, TGP indirectly shields melanocytes from inflammation-mediated damage.

In ACD and psoriasis, the primary mechanism of TGP is enhances cellular antioxidant capacity and consequently reduces inflammation. TGP further inhibits ERK, JNK, and p38 in the MAPKs pathway, thereby diminishing oxidative stress-induced inflammatory responses (Yu J. H. et al., 2017). Moreover, TGP can promote the expression of antioxidant genes, suppress excessive proliferation of keratinocytes, and reduce inflammatory cell infiltration, thus alleviating the pathological progression of these diseases (Wang and Wang, 2021). Additionally, in psoriasis and other Th17 cell-dominant dermatologic diseases, TGP mitigates inflammation by regulating the STAT signaling pathway (Li B. B. et al., 2019).

### 9.3 Immunomodulation

In addition to the two previously mentioned pharmacological effects, TGP demonstrates significant immunomodulatory properties in the treatment of urticaria, SS, OLP, SLE, and vitiligo (Zhao et al., 2024). In urticaria, TGP mitigates allergic reactions and itching by inhibiting the NF-κB signaling pathway, which reduces the release of pro-inflammatory cytokines and histamine (Long et al., 2010). In SS, TGP helps regulate the Th1/Th2 balance and inhibits the STAT signaling pathway, thereby reducing glandular inflammation. It also enhances the function of regulatory T cells, contributing to the maintenance of immune tolerance (Zhang et al., 2015). Furthermore, in OLP, TGP suppresses abnormal T-cell activation and reduces mucosal inflammation by inhibiting the NF-κB and MAPKs pathways, thereby decreasing the release of pro-inflammatory cytokines and alleviating oxidative stress-induced tissue damage (Wang et al., 2016). In SLE, TGP exerts its immunomodulatory effects primarily by inhibiting the overexpression of inflammatory cytokines such as TNF-α, IL-1β, and IL-6, while regulating immune cell activity. This involves suppressing the hyperactivity of T and B cells and promoting the differentiation of regulatory T cells to restore immune homeostasis (Tu et al., 2019). Additionally, by inhibiting the activation of the NF-κB and MAPKs pathways, TGP reduces the release of pro-inflammatory cytokines, thereby alleviating inflammation and providing protective effects in SLE.

In vitiligo, TGP modulates T-cell activity by inhibiting CD8^+^ T-cell attacks on melanocytes, thereby reducing melanocyte destruction (Zhu et al., 2014). Furthermore, TGP activates the PI3K/Akt signaling pathway to enhance melanocyte survival, while also inhibiting the NF-κB pathway to reduce pro-inflammatory cytokine release and inflammation-induced melanocyte damage (Di Bartolomeo et al., 2023). These mechanisms highlight the critical role of TGP in regulating immune responses across a range of immune-mediated dermatologic diseases.

## 10 Problems with current research and directions for future research

### 10.1 Problems with current research

Research on TGP in the treatment of dermatologic diseases has demonstrated significant anti-inflammatory, immunomodulatory, and antioxidant effects, but there are several limitations. Most studies are based on animal models and *in vitro* experiments, with small sample sizes that limit statistical power and make the findings susceptible to external objective factors, further affecting their reproducibility and representativeness. Additionally, variations in the purity and composition of TGP extracts used in different studies further complicate the consistency of the results. Current research lacks sufficient focus on the long-term toxicity and safety of low-dose chronic administration, especially in cases of prolonged or high-dose use, which impacts the risk assessment for clinical applications. Furthermore, the primary active metabolite of TGP, paeoniflorin, has low oral bioavailability and is rapidly metabolized, limiting its therapeutic efficacy. While most research has focused on the overall effects of TGP and PF, the pharmacokinetics, pharmacodynamics, and potential synergistic effects of other metabolites such as albiflorin and oxypaeoniflorin remain largely unexplored.

Clinical trials on TGP also face certain challenges. Although TGP has shown some efficacy in treating dermatological conditions, existing clinical trials are small in scale and lack external validity (Chen et al., 2019). The absence of large-scale, long-term randomized controlled trials limits the thorough validation of TGP’s therapeutic efficacy and safety in human populations. Moreover, studies on the dose-response relationship, long-term safety, and the safety of TGP use in specific populations (e.g., pregnant women, children, and the elderly) remain insufficient. Some patients have reported mild gastrointestinal discomfort and skin allergies during clinical use, which, although generally mild, may affect patient compliance. Therefore, to ensure the safe and effective clinical application of TGP, larger-scale, systematic research is urgently needed to provide more reliable evidence.

### 10.2 Directions for future research

Future research should focus on addressing these critical challenges to fully realize the therapeutic potential of TGP in dermatology. Large-scale, high-quality clinical trials are imperative to validate TGP’s efficacy and safety across various dermatologic conditions, enabling a comprehensive evaluation of its therapeutic applications. Additionally, modern multi-omics approaches, including genomics, transcriptomics, and proteomics, should be employed to investigate the molecular mechanisms underlying TGP’s actions (Cao et al., 2024), particularly its interactions with immune systems, inflammatory pathways, and oxidative stress responses. These studies could elucidate the specific cellular and molecular interactions mediated by TGP, further enhancing our understanding of its mechanisms of action. Another crucial area for future research is the optimization of TGP’s drug delivery systems and formulations. Given its low oral bioavailability, the development of topical delivery systems may significantly improve therapeutic outcomes. Innovative formulation designs, such as nanoparticles and transdermal delivery systems, offer promising avenues to enhance bioavailability and expand TGP’s applications in dermatologic treatments. Such advancements could also reduce the required dosage, thereby minimizing adverse effects, improving safety, and enhancing patient compliance (Kalepu and Nekkanti, 2015; Wang, 2018; Parra et al., 2021). Equally important is the standardization of TGP’s content and bioactive components. Ensuring consistent quality and precise quantification of these active constituents is vital for achieving reproducible results, optimizing therapeutic efficacy, and facilitating broader clinical acceptance. In summary, future research should prioritize several key areas, including clinical trial design, mechanistic exploration, pharmacokinetic studies, and formulation optimization. By addressing these aspects, a more comprehensive and reliable scientific foundation can be established for the application of TGP in dermatology. With these advancements, TGP holds significant promise for expanded use in the treatment of dermatologic diseases.

## 11 Conclusion

Dermatologic diseases have long been a central subject of academic study because of their serious threat to human health and their influence on quality of life. *Paeonia lactiflora Pall.*, widely cultivated and utilized for its beautiful flowers and medicinal value, has garnered significant attention. In traditional Chinese medicine, its primary effects include nourishing the blood and soothing the liver, astringing yin to stop sweating, relieving pain by easing tension, and calming hyperactive liver yang. TGP, a group of active metabolites extracted from *Paeonia lactiflora Pall.*, exhibits a range of pharmacological properties, including anti-inflammatory, analgesic, immunomodulatory, and antioxidant effects, as well as organ protection and protective actions on the cardiovascular and nervous systems. These multifaceted properties provide significant therapeutic potential in the treatment of dermatologic diseases. In conclusion, TGP offers extensive pharmacological benefits with minimal adverse effects and excellent tolerance, underscoring its safety for clinical use. With further in-depth, systematic, and standardized research, TGP holds promise as an innovative approach to addressing more complex dermatological conditions, maximizing the strengths of traditional Chinese medicine to improve the lives of countless patients.

## References

[B1] Adly M. Woo T. E. Traboulsi D. Klassen D. Hardin J. (2021). Understanding dermatologic concerns among persons experiencing homelessness: a scoping review and discussion for improved delivery of care. J. Cutan. Med. Surg. 25 (6), 616–626. 10.1177/12034754211004558 33818163 PMC8640276

[B2] Ahn S. S. Lee Y. H. Yeo H. Jung E. Lim Y. Shin S. Y. (2022). Saikosaponin A and saikosaponin C reduce TNF-α-induced TSLP expression through inhibition of MAPK-mediated EGR1 expression in HaCaT keratinocytes. Int. J. Mol. Sci. 23 (9), 4857. 10.3390/ijms23094857 35563251 PMC9105331

[B3] Alexopoulou L. (2022). Nucleic acid-sensing toll-like receptors: important players in Sjogren's syndrome. Front. Immunol. 13, 980400. 10.3389/fimmu.2022.980400 36389822 PMC9659959

[B4] Aloui A. Maoua M. El Guedri S. Moussa A. Bouhoula M. Chouchene A. (2022). Contribution of patch tests with occupational handled products in the diagnosis of occupational contact dermatitis: a 10-year review. Dermatology Res. Pract. 2022, 6768932. 10.1155/2022/6768932 PMC9377969 35979389

[B5] Bartko E. A. Elberling J. Blom L. H. Poulsen L. K. Jensen B. M. (2023). Elevated, FcεRI‐dependent MRGPRX2 expression on basophils in chronic urticaria. Skin Health Dis. 3 (3), e195. 10.1002/ski2.195 37275407 PMC10233071

[B6] Bracken S. J. Abraham S. MacLeod A. S. (2019). Autoimmune theories of chronic spontaneous urticaria. Front. Immunol. 10, 627. 10.3389/fimmu.2019.00627 30984191 PMC6450064

[B7] Cao J. X. Li C. F. Cui Z. Deng S. W. Lei T. Liu W. (2024). Spatial transcriptomics: a powerful tool in disease understanding and drug discovery. Theranostics 14 (7), 2946–2968. 10.7150/thno.95908 38773973 PMC11103497

[B8] Chandel H. S. Pathak A. K. Tailang M. (2011). Standardization of some herbal antidiabetic drugs in polyherbal formulation. Pharmacogn. Res. 3 (1), 49–56. 10.4103/0974-8490.79116 PMC3119272 21731396

[B9] Chen F. Hu Y. Xie Y. T. Zhao Z. H. Ma L. Li Z. L. (2020). Total glucosides of paeony alleviate cell apoptosis and inflammation by targeting the long noncoding RNA XIST/MicroRNA-124-3p/ITGB1 Axis in renal ischemia/reperfusion injury. Mediat. Inflamm. 13, 8869511. 10.1155/2020/8869511 PMC7710434 33299380

[B10] Chen T. Fu L. X. Zhang L. W. Yin B. Zhou P. M. Cao N. (2016). Paeoniflorin suppresses inflammatory response in imiquimod-induced psoriasis-like mice and peripheral blood mononuclear cells (PBMCs) from psoriasis patients. Can. J. Physiology Pharmacol. 94 (8), 888–894. 10.1139/cjpp-2015-0483 27348512

[B11] Chen Y. Wang Y. F. Song S. S. Zhang X. L. Wu L. L. Wu J. B. (2023). Topical application of baicalin combined with echinacoside ameliorates psoriatic skin lesions by suppressing the inflammation-related TNF signaling pathway and the angiogenesis-related VEGF signaling pathway. Acs Omega 8 (43), 40260–40276. 10.1021/acsomega.3c04281 37929119 PMC10620902

[B12] Chen Y. F. Wang L. D. Cao Y. Li N. N. (2022). Total glucosides of Paeonia lactiflora for safely reducing disease activity in systemic lupus erythematosus: a systematic review and meta-analysis. Front. Pharmacol. 13, 834947. 10.3389/fphar.2022.834947 35173622 PMC8841895

[B13] Chen Y. Y. Wang Y. Xu L. Zhu W. N. Xu C. S. Xu M. M. (2019). Influence of total glucosides of paeony on PD-1/PD-L1 expression in primary Sjogren's syndrome. Int. J. Rheumatic Dis. 22 (2), 200–206. 10.1111/1756-185x.13391 30338648

[B14] Chimenti M. S. Sunzini F. Fiorucci L. Botti E. Fonti G. L. Conigliaro P. (2018). Potential role of cytochrome c and tryptase in psoriasis and psoriatic arthritis pathogenesis: focus on resistance to apoptosis and oxidative stress. Front. Immunol. 9, 2363. 10.3389/fimmu.2018.02363 30429845 PMC6220124

[B15] Cintosun A. Lara-Corrales I. Pope E. (2020). Mechanisms of cannabinoids and potential applicability to skin diseases. Clin. Drug Investig. 40 (4), 293–304. 10.1007/s40261-020-00894-7 32060787

[B16] Claveau J. Lavoie A. Brunet C. Bedard P. M. Hebert J. (1993). Chronic idiopathic urticaria: possible contribution of histamine-releasing factor to pathogenesis. J. allergy Clin. Immunol. 92 (1 Pt 1), 132–137. 10.1016/0091-6749(93)90047-j 7687607

[B17] Dawid-Pac R. (2013). Medicinal plants used in treatment of inflammatory skin diseases. Postepy Dermatol. I Alergol. 30 (3), 170–177. 10.5114/pdia.2013.35620 PMC3834722 24278070

[B18] Deng G. Ye W. G. Wan Q. Wang J. L. (2020). Effectiveness of moxibustion therapy in the treatment of urticaria A protocol for a systematic review and meta-analysis. Medicine 99 (49), e23481. 10.1097/md.0000000000023481 33285750 PMC7717810

[B19] Deng H. Yan C. L. Xiao T. Yuan D. F. Xu J. H. (2010). Total glucosides of *Paeonia lactiflora* Pall inhibit vascular endothelial growth factor-induced angiogenesis. J. Ethnopharmacol. 127 (3), 781–785. 10.1016/j.jep.2009.09.053 19914370

[B20] Deng J. Pan W. Y. Ji N. Liu N. Chen Q. Chen J. H. (2022). Cell-free DNA promotes inflammation in patients with oral lichen planus via the STING pathway. Front. Immunol. 13, 838109. 10.3389/fimmu.2022.838109 35493447 PMC9049180

[B21] Di Bartolomeo L. Custurone P. Irrera N. Borgia F. Vaccaro F. Squadrito F. (2023). Vitiligo and mental health: natural compounds' usefulness. Antioxidants 12 (1), 176. 10.3390/antiox12010176 36671038 PMC9854903

[B22] Didona D. Hertl M. (2022). Detection of anti-desmoglein antibodies in oral lichen planus: what do we know so far. Front. Immunol. 13, 1001970. 10.3389/fimmu.2022.1001970 36263026 PMC9575987

[B23] Donetti E. Sommariva M. Chiricozzi A. Prignano F. (2023). Editorial: the immunobiology of the skin in response to the environment. Front. Immunol. 14, 1220335. 10.3389/fimmu.2023.1220335 37283764 PMC10240051

[B24] Drake L. Reyes-Hadsall S. Martinez J. Heinrich C. Huang K. T. Mostaghimi A. (2023). Evaluation of the safety and effectiveness of nutritional supplements for treating hair loss A systematic review. Jama Dermatol. 159 (1), 79–86. 10.1001/jamadermatol.2022.4867 36449274

[B25] Du Z. W. Wang H. P. Gao Y. Zheng S. M. Kou X. L. Sun G. Q. (2023). Exploring the potential molecular mechanism of sijunzi decoction in the treatment of non-segmental vitiligo based on network pharmacology and molecular docking. Clin. Cosmet. Investigational Dermatology 16, 821–836. 10.2147/ccid.S403732 PMC10075956 37033783

[B26] Ezzedine K. Eleftheriadou V. Whitton M. van Geel N. (2015). Vitiligo. Lancet 386 (9988), 74–84. 10.1016/s0140-6736(14)60763-7 25596811

[B27] Fang Q. L. Li Q. L. Zhong L. K. Qi Y. J. Xin W. X. Fang L. (2023). Paeony attenuates high fat diet-induced kidney injury via inflammation inhibition. Pak. J. Pharm. Sci. 36 (4), 1217–1225. 10.36721/pjps.2023.36.4.Reg.1217-1225.1 37599498

[B28] Fattah N. Darwish Y. W. (2013). *In vitro* antibiotic susceptibility patterns of Propionibacterium acnes isolated from acne patients: an Egyptian university hospital-based study. J. Eur. Acad. Dermatology Venereol. 27 (12), 1546–1551. 10.1111/jdv.12057 23279041

[B29] Feldo M. Wójciak M. Ziemlewska A. Dresler S. Sowa I. (2022). Modulatory effect of diosmin and diosmetin on metalloproteinase activity and inflammatory mediators in human skin fibroblasts treated with lipopolysaccharide. Molecules 27 (13), 4264. 10.3390/molecules27134264 35807509 PMC9268213

[B30] Feng Z. Zhang B. Q. Zhu Y. M. Yu B. B. Fu L. Zhou L. L. (2019). The effectiveness and safety of total glucosides of paeony in primary sjogren's syndrome: a systematic review and meta-analysis. Front. Pharmacol. 10, 550. 10.3389/fphar.2019.00550 31178729 PMC6543198

[B31] Fontenot J. D. Gavin M. A. Rudensky A. Y. (2003). Foxp3 programs the development and function of CD4^+^CD25^+^ regulatory T cells. Nat. Immunol. 4 (4), 330–336. 10.1038/ni904 12612578

[B32] Fowler J. E. Woolery-Lloyd H. Waldorf H. Saini R. (2010). Innovations in natural ingredients and their use in skin care. J. Drugs Dermatology 9 (6), S72–s83. 20626172

[B33] Gangwar V. Garg A. Lomore K. Korla K. Bhat S. S. Rao R. (2021). Immunomodulatory effects of a concoction of natural bioactive compounds-mechanistic insights. Biomedicines 9 (11), 1522. 10.3390/biomedicines9111522 34829751 PMC8615223

[B34] Geng L. Zhang X. M. Tang Y. Gu W. L. (2022). Identification of potential key biomarkers and immune infiltration in oral lichen planus. Dis. Markers 20, 7386895. 10.1155/2022/7386895 PMC8898126 35256894

[B35] Gobbo M. Rupel K. Zoi V. Perinetti G. Ottaviani G. Di Lenarda R. (2017). Scoring systems for Oral Lichen Planus used by differently experienced raters. Med. Oral Patol. Oral Y Cirugia Bucal 22 (5), E562–E571. 10.4317/medoral.21833 PMC5694178 28809373

[B36] Gong X. H. Li H. Guo H. T. Wu S. W. Lu C. Q. Chen Y. M. (2022). Efficacy and safety of total glucosides of paeony in the treatment of systemic lupus erythematosus: a systematic review and meta-analysis. Front. Pharmacol. 13, 932874. 10.3389/fphar.2022.932874 36569311 PMC9768345

[B37] Gu X. Y. Li Z. R. Su J. (2024). Air pollution and skin diseases: a comprehensive evaluation of the associated mechanism. Ecotoxicol. Environ. Saf. 278, 116429. 10.1016/j.ecoenv.2024.116429 38718731

[B38] Gui J. C. Li Z. M. Zhou B. Li Q. Zhang Y. Wang Y. J. (2024). Combination of total glucosides of paeony, narrow-band ultraviolet B, and oral corticosteroid mini-pulse therapy for nonsegmental vitiligo: a retrospective study. Skin Res. Technol. 30 (6), e13769. 10.1111/srt.13769 38887837 PMC11182781

[B39] Guo J. Peng L. Zeng J. H. Zhang M. H. Xu F. Zhang X. T. (2021a). Paeoniflorin suppresses allergic and inflammatory responses by promoting autophagy in rats with urticaria. Exp. Ther. Med. 21 (6), 590. 10.3892/etm.2021.10022 33884028 PMC8056118

[B40] Guo X. R. Yang X. R. Li Q. Shen X. Y. Zhong H. Y. Yang Y. (2021b). The microbiota in systemic lupus erythematosus: an update on the potential function of probiotics. Front. Pharmacol. 12, 759095. 10.3389/fphar.2021.759095 34887760 PMC8650621

[B41] Guo Y. X. Mao W. Y. Bai N. N. Jin L. Tang S. Y. Lin X. C. (2024). Integrated network pharmacological analysis revealed that Smilax glabra Roxb. alleviates IMQ-induced psoriatic skin inflammation through regulating T cell immune response. J. Ethnopharmacol. 325, 117836. 10.1016/j.jep.2024.117836 38301985

[B42] Harrington L. E. Hatton R. D. Mangan P. R. Turner H. Murphy T. L. Murphy K. M. (2005). Interleukin 17-producing CD4^+^ effector T cells develop via a lineage distinct from the T helper type 1 and 2 lineages. Nat. Immunol. 6 (11), 1123–1132. 10.1038/ni1254 16200070

[B43] He K. Wang Z. Y. Liu M. Du W. Q. Yin T. Y. Bai R. M. (2024). Exploring the effect of xiao-chai-hu decoction on treating psoriasis based on network pharmacology and experiment validation. Curr. Pharm. Des. 30 (3), 215–229. 10.2174/0113816128288527240108110844 38532341

[B44] He Y. Yang Y. Liao Y. Li C. Xiong X. (2016). A clinical study of levocetirizine combined with montelukast and total glucosides of paeony in treating 44 cases of chronic spontaneous urticaria. Chin. J. Dermatovenereology 30 (9), 985–987.

[B45] Homey B. Bunemann E. (2004). Chemokines and inflammatory skin diseases. Ernst Schering Res. Found. workshop 45, 69–83. 10.1007/978-3-662-05403-1_6 14699795

[B46] Hongying Z. Tongxin S. H. I. Chunyang L. I. (2011). Effects of total glucosides of paeony on cell proliferation of and expression of vascular endothelial growth factor (VEGF)and interleukin (IL)-23 in human HaCaT keratinocytes. Chin. Journa Dermatology 44 (5), 343–346.

[B47] Hori S. Nomura T. Sakaguchi S. (2003). Control of regulatory T cell development by the transcription factor Foxp3. Science 299 (5609), 1057–1061. 10.1126/science.1079490 12522256

[B48] Hu M. R. Chen C. G. Liu J. J. Cai L. Shao J. Y. Chen Z. X. (2020). The melanogenic effects and underlying mechanism of paeoniflorin in human melanocytes and vitiligo mice. Fitoterapia 140, 104416. 10.1016/j.fitote.2019.104416 31704261

[B49] Hu W. Zhang J. Z. Wang H. J. Guan M. M. Dai L. H. Li J. (2023). Protective effects of isorhamnetin against H_2_O_2_-induced oxidative damage in HaCaT cells and comprehensive analysis of key genes. Sci. Rep. 13 (1), 2498. 10.1038/s41598-023-27575-7 36781904 PMC9925802

[B50] Huang Y. Wang H. Chen Z. Wang Y. Qin K. Huang Y. (2019). Synergistic and hepatoprotective effect of total glucosides of paeony on ankylosing spondylitis: a systematic review and meta-analysis. Front. Pharmacol. 10, 231. 10.3389/fphar.2019.00231 30941036 PMC6433937

[B51] Jakimiuk K. Tomczyk M. (2024). A review of the traditional uses, phytochemistry, pharmacology, and clinical evidence for the use of the genus Alchemilla (Rosaceae). J. Ethnopharmacol. 320, 117439. 10.1016/j.jep.2023.117439 37981119

[B52] Jalel A. Hamdaoui M. H. (2009). Study of total antioxidant status and glutathione peroxidase activity in Tunisian vitiligo patients. Indian J. dermatology 54 (1), 13–16. 10.4103/0019-5154.48978 PMC2800862 20049261

[B53] Jiang H. J. Li J. Wang L. Wang S. J. Nie X. Chen Y. (2020). Total glucosides of paeony: a review of its phytochemistry, role in autoimmune diseases, and mechanisms of action. J. Ethnopharmacol. 258, 112913. 10.1016/j.jep.2020.112913 32371143

[B54] Jiang T. Guo J. Wang Y. Wu H. Chen Y. Wang S. (2023). Total glucosides of paeony alleviates experimental Sjögren's syndrome through inhibiting NLRP3 inflammasome activation of submandibular gland cells. Clin. Exp. Rheumatology 41 (12), 2502–2510. 10.55563/clinexprheumatol/7kbuok 38149512

[B55] Jin B. Sun T. Yu X. H. Yang Y. X. Yeo A. E. T. (2012). The effects of TLR activation on T-cell development and differentiation. Clin. and Dev. Immunol. 32, 836485. 10.1155/2012/836485 PMC3376488 22737174

[B56] Jin Y. Z. Zhang A. C. (2022). Total glucosides of paeony ameliorates oxidative stress, apoptosis and inflammatory response by regulating the Smad7‑TGF‑β pathway in allergic rhinitis. Mol. Med. Rep. 25 (3), 83. 10.3892/mmr.2022.12599 35029288 PMC8778736

[B57] Kalepu S. Nekkanti V. (2015). Insoluble drug delivery strategies: review of recent advances and business prospects. Acta Pharm. Sin. B 5 (5), 442–453. 10.1016/j.apsb.2015.07.003 26579474 PMC4629443

[B58] Khan D. A. (2013). Alternative agents in refractory chronic urticaria: evidence and considerations on their selection and use. J. Allergy Clin. Immunology-in Pract. 1 (5), 433–440.e1. 10.1016/j.jaip.2013.06.003 24565613

[B59] Kim W. B. Jerome D. Yeung J. (2017). Diagnosis and management of psoriasis. Can. Fam. Physician 63 (4), 278–285. 28404701 PMC5389757

[B60] Kimber I. Travis M. A. Martin S. F. Dearman R. J. (2012). Immunoregulation of skin sensitization and regulatory T cells. Contact Dermat. 67 (4), 179–183. 10.1111/j.1600-0536.2012.02148.x 22804346

[B61] Kitsioulis N. A. Xepapadaki P. Roussaki-Schulze A. V. Papadopoulos N. Zafiriou E. (2017). Effectiveness of autologous whole-blood injections in patients with refractory chronic spontaneous urticaria. Int. Archives Allergy Immunol. 172 (3), 161–166. 10.1159/000458152 28380487

[B62] Kong L. W. Wang S. S. Wu X. Zuo F. G. Qin H. H. Wu J. F. (2016). Paeoniflorin attenuates ultraviolet B-induced apoptosis in human keratinocytes by inhibiting the ROS-p38-p53 pathway. Mol. Med. Rep. 13 (4), 3553–3558. 10.3892/mmr.2016.4953 26936104

[B63] Kular L. Pakradouni J. Kitabgi P. Laurent M. Martinerie C. (2011). The CCN family: a new class of inflammation modulators? Biochimie 93 (3), 377–388. 10.1016/j.biochi.2010.11.010 21130134

[B64] Lei D. Bo W. (2019). Effect of total glucosides of paeony on interleukins 18 and 33 MRNA expressions in patients with allergic contact dermatitis. Acta Medica Mediterr. 35 (2), 709–713. 10.19193/0393-6384_2019_2_106

[B65] Lei M. J. Bai F. Zhang Q. Y. Yang Q. Q. Tian Z. (2023). Total glucosides of paeony regulate immune imbalance mediated by dermal mesenchymal stem cells in psoriasis mice. Chin. J. Integr. Med. 29 (6), 517–525. 10.1007/s11655-023-3737-y 37222920

[B66] Li B. B. He S. C. Liu R. Huang L. L. Liu G. Wang R. X. (2019a). Total glucosides of paeony attenuates animal psoriasis induced inflammatory response through inhibiting STAT1 and STAT3 phosphorylation. J. Ethnopharmacol. 243, 112121. 10.1016/j.jep.2019.112121 31356966

[B67] Li B. B. Liu G. Liu R. He S. C. Li X. Huang L. L. (2020a). Total glucosides of paeony (TGP) alleviates Sjogren's syndrome through inhibiting inflammatory responses in mice. Phytomedicine 71, 153203. 10.1016/j.phymed.2020.153203 32402913

[B68] Li C. L. He J. Li Z. G. Zheng L. W. Hua H. (2013). Effects of total glucosides of paeony for delaying onset of Sjogren's syndrome: an animal study. J. Cranio-Maxillofacial Surg. 41 (7), 610–615. 10.1016/j.jcms.2012.11.042 23333492

[B69] Li H. Sun X. Zhang J. Sun Y. Huo R. Li H. (2016a). Paeoniflorin ameliorates symptoms of experimental Sjogren's syndrome associated with down-regulating Cyr61 expression. Int. Immunopharmacol. 30, 27–35. 10.1016/j.intimp.2015.11.023 26630293

[B70] Li M. Li Y. Xiang L. J. Li L. F. (2022). Efficacy and safety of total glucosides of paeony as an add-on treatment in adolescents and adults with chronic urticaria: a systematic review and meta-analysis. Front. Pharmacol. 13, 961371. 10.3389/fphar.2022.961371 36263138 PMC9574670

[B71] Li M. Y. Jiang A. P. (2019). DNA methylation was involved in total glucosides of paeony regulating ERα for the treatment of female systemic lupus erythematosus mice. J. Pharmacol. Sci. 140 (2), 187–192. 10.1016/j.jphs.2019.07.003 31345653

[B72] Li S. Bai J. Z. Fan G. F. Liu R. P. (2023). Total glucosides of paeony alleviates scleroderma by inhibiting type I interferon responses. J. Ethnopharmacol. 302, 115897. 10.1016/j.jep.2022.115897 36334818

[B73] Li T. T. Zhou D. M. Xu X. Y. Qu J. H. Jiang C. Y. Lan H. B. (2019b). Effect of Traditional Chinese Medicine plus narrow-band medium-wave ultraviolet B radiation on moderate-to-severe psoriasis vulgaris in a case series. J. Traditional Chin. Med. 39 (5), 692–699. 32186119

[B74] Li X. Li Z. Li X. (2016b). Curative effect of total glucosides of paeony capsule combined with narrow-band ultraviolet B(NB-UVB) in the treatment of psoriasis and the effect to T cell subset level. Chin. J. Dermatovenereology 30 (11), 1205–1207.

[B75] Li Y. Liu S. Chang Y. Wang T. Li Z. Wang J. (2016c). Curative effect of total glucosides of paeony combined with wolfberry in the treatment of olp. J. Oral Sci. Res. 32 (8), 861–864.

[B76] Li Z. Yang J. J. Xie Q. B. (2018). Clinical study on Total Glucosides of Paeony Capsules combined with tacrolimus in treatment of systemic lupus erythematosus. Drugs&Clinic 33 (06), 1513–1517.

[B77] Li Z. Zhou Q. Zhang G. Zhang P. (2020b). Clinical efficacy of shashen maidongtang plus total glucosides of paeony capsule in treatment of primary sjogren's syndrome with based on theory of fluid metabolism. Chin. J. Exp. Traditional Med. Formulae 26 (20), 100–104.

[B78] Liao Y. H. Sun L. X. Nie M. F. Li J. C. Huang X. F. Heng S. J. (2023). Modulation of skin inflammatory responses by aluminum adjuvant. Pharmaceutics 15 (2), 576. 10.3390/pharmaceutics15020576 36839900 PMC9966661

[B79] Lim S. S. Shin K. Mun J.-H. (2022). Dermoscopy for cutaneous fungal infections: a brief review. Health Sci. Rep. 5 (1), e464. 10.1002/hsr2.464 35024456 PMC8733849

[B80] Lin J. P. Xiao L. B. Ouyang G. L. Shen Y. Huo R. F. Zhou Z. (2012). Total glucosides of paeony inhibits Th1/Th17 cells via decreasing dendritic cells activation in rheumatoid arthritis. Cell. Immunol. 280 (2), 156–163. 10.1016/j.cellimm.2012.12.005 23399842

[B81] Liu S. Li Y. Wu C. J. (2023). Paeoniflorin suppresses the apoptosis and inflammation of human coronary artery endothelial cells induced by oxidized low-density lipoprotein by regulating the Wnt/β-catenin pathway. Pharm. Biol. 61 (1), 1454–1461. 10.1080/13880209.2023.2220360 37674320 PMC10486282

[B82] Liu W. Z. He M. J. Long L. Mu D. L. Xu M. S. Xing X. (2014). Interferon-γ and interleukin-4 detected in serum and saliva from patients with oral lichen planus. Int. J. Oral Sci. 6 (1), 22–26. 10.1038/ijos.2013.74 24158143 PMC3967304

[B83] Liu X. Li X. M. Li X. P. Li Z. J. Zhao D. B. Liu S. Y. (2019). The efficacy and safety of total glucosides of peony in the treatment of primary Sjogren's syndrome: a multi-center, randomized, double-blinded, placebo-controlled clinical trial. Clin. Rheumatol. 38 (3), 657–664. 10.1007/s10067-018-4315-8 30280368

[B84] Liu Y. Y. Su X. R. Liu S. S. Yang S. S. Jiang C. Y. Zhang Y. (2017). Zebrafish phosvitin-derived peptide Pt5 inhibits melanogenesis via cAMP pathway. Fish Physiology Biochem. 43 (2), 517–525. 10.1007/s10695-016-0306-3 28130732

[B85] Long J. W. Wang Y. Y. Pi X. M. Tu Y. T. (2010). Clinical observation on the treatment of chronic urticaria with total glucosides of paeony capsule combined with citirizine. Chin. J. Integr. Med. 16 (4), 353–356. 10.1007/s11655-010-0504-2 20697948

[B86] Lu Y. S. Jiang Y. Yuan J. P. Jiang S. B. Yang Y. Zhu P. Y. (2020). UVA induced oxidative stress was inhibited by paeoniflorin/nrf2 signaling or PLIN2. Front. Pharmacol. 11, 736. 10.3389/fphar.2020.00736 32499710 PMC7243259

[B87] Luo Y. Hara T. Kawashima A. Ishido Y. Suzuki S. Ishii N. (2020). Pathological role of excessive DNA as a trigger of keratinocyte proliferation in psoriasis. Clin. Exp. Immunol. 202 (1), 1–10. 10.1111/cei.13455 32415989 PMC7586253

[B88] Mummed B. Abraha A. Feyera T. Nigusse A. Assefa S. (2018). *In vitro* antibacterial activity of selected medicinal plants in the traditional treatment of skin and wound infections in eastern Ethiopia. BioMed Res. Int. 2018, 1862401. 10.1155/2018/1862401 30079345 PMC6069697

[B89] Orsmond A. Bereza-Malcolm L. Lynch T. March L. Xue M. L. (2021). Skin barrier dysregulation in psoriasis. Int. J. Mol. Sci. 22 (19), 10841. 10.3390/ijms221910841 34639182 PMC8509518

[B90] Ouchi T. Nakato G. Udey M. C. (2016). EpCAM expressed by murine epidermal Langerhans cells modulates immunization to an epicutaneously applied protein antigen. J. Investigative Dermatology 136 (8), 1627–1635. 10.1016/j.jid.2016.04.005 PMC4958526 27106675

[B91] Pandey R. Al-Nuaimi Y. Mishra R. K. Spurgeon S. K. Goodfellow M. (2021). Role of subnetworks mediated by TNFα, IL-23/IL-17 and IL-15 in a network involved in the pathogenesis of psoriasis. Sci. Rep. 11 (1), 13. 10.1038/s41598-020-80507-7 33500449 PMC7838322

[B92] Pang Y. B. Wu S. He Y. J. Nian Q. Lei J. Yao Y. J. (2021). Plant-derived compounds as promising therapeutics for vitiligo. Front. Pharmacol. 12, 685116. 10.3389/fphar.2021.685116 34858164 PMC8631938

[B93] Park S. Y. Lee H. J. Kim S. H. Kim S. B. Choi Y. H. Kim Y. K. (2018). Factors affecting treatment outcomes in patients with oral lichen planus lesions: a retrospective study of 113 cases. J. Periodontal Implant Sci. 48 (4), 213–223. 10.5051/jpis.2018.48.4.213 30202605 PMC6125672

[B94] Parra A. Jarak I. Santos A. Veiga F. Figueiras A. (2021). Polymeric micelles: a promising pathway for dermal drug delivery. Materials 14 (23), 7278. 10.3390/ma14237278 34885432 PMC8658125

[B95] Patel P. Wang J. Y. Mineroff J. Jagdeo J. (2023). Evaluation of curcumin for dermatologic conditions: a systematic review. Archives Dermatological Res. 316 (1), 37. 10.1007/s00403-023-02754-8 38085369

[B96] Peng L. Ma Z. Chu W. H. Jiang P. S. Fu Y. Q. Wang P. (2023). Identification and hepatoprotective activity of total glycosides of paeony with high content of paeoniflorin extracted from Paeonia lactiflora Pall. Food Chem. Toxicol. 173, 113624. 10.1016/j.fct.2023.113624 36681265

[B97] Peng L. Wen L. J. Zhang J. Zhang X. T. Wei Q. Guo J. (2022). Circadian pharmacological effects of paeoniflorin on mice with urticaria-like lesions. Front. Pharmacol. 12, 639580. 10.3389/fphar.2021.639580 35222003 PMC8863972

[B98] Perl A. (2012). Oxidative stress and endosome recycling are complementary mechanisms reorganizing the T-cell receptor signaling complex in SLE. Clin. Immunol. 142 (3), 219–222. 10.1016/j.clim.2011.12.011 22245265 PMC4048946

[B99] Popov A. Mirkov I. Miljkovic D. Belij S. Zolotarevski L. Kataranovski D. (2011). Contact allergic response to dinitrochlorobenzene (DNCB) in rats: insight from sensitization phase. Immunobiology 216 (7), 763–770. 10.1016/j.imbio.2010.12.007 21281978

[B100] Pourang A. Hendricks A. J. Shi V. Y. (2020). Managing dermatology patients who prefer “all natural” treatments. Clin. Dermatology 38 (3), 348–353. 10.1016/j.clindermatol.2019.10.025 32563348

[B101] Pu J. B. Zheng J. X. Liang W. Q. Hu Y. J. (2011). Determination of paeoniflorin and total saponin in Paeonia lactiflora extract. Chin. J. Traditional Med. Sci. Technol. 18 (04), 326–327.

[B102] Qiao Z. H. Wang X. X. Xiang L. H. Zhang C. F. (2016). Dysfunction of autophagy: a possible mechanism involved in the pathogenesis of vitiligo by breaking the redox balance of melanocytes. Oxidative Med. Cell. Longev. 7, 3401570. 10.1155/2016/3401570 PMC5153471 28018522

[B103] Qing M. Yang D. Shang Q. Peng J. Deng J. Lu J. (2023). CD8^+^ tissue-resident memory T cells induce oral lichen planus erosion via cytokine network. eLife 12, e83981. 10.7554/eLife.83981 37555396 PMC10465124

[B104] Qiu J. Chen M. Liu J. Huang X. Chen J. Zhou L. (2016). The skin-depigmenting potential of Paeonia lactiflora root extract and paeoniflorin: *in vitro* evaluation using reconstructed pigmented human epidermis. Int. J. Cosmet. Sci. 38 (5), 444–451. 10.1111/ics.12309 26826350

[B105] Qiu W. L. Yu T. Deng G. M. (2022). The role of organ-deposited IgG in the pathogenesis of multi-organ and tissue damage in systemic lupus erythematosus. Front. Immunol. 13, 924766. 10.3389/fimmu.2022.924766 36311714 PMC9609414

[B106] Rajasekar S. Park D. J. Park C. Park S. Park Y. H. Kim S. T. (2012). *In vitro* and *in vivo* anticancer effects of *Lithospermum erythrorhizon* extract on B16F10 murine melanoma. J. Ethnopharmacol. 144 (2), 335–345. 10.1016/j.jep.2012.09.017 22995444

[B107] Ramaswamy S. Kuppuswamy G. Dwarampudi L. P. Kadiyala M. Menta L. Kannan E. (2014). Development and validation of simultaneous estimation method for curcumin and piperine by RP-UFLC. Pak. J. Pharm. Sci. 27 (4), 901–906. 25015458

[B108] Ranneh Y. Ali F. Al-Qubaisi M. Esa N. M. Ismail A. (2016). The inhibitory activity of cocoa phenolic extract against pro-inflammatory mediators secretion induced by lipopolysaccharide in RAW 264.7 cells. Springerplus 5, 547. 10.1186/s40064-016-2138-0 27190746 PMC4850146

[B109] Reich K. Garbe C. Blaschke V. Maurer C. Middel P. Westphal G. (2001). Response of psoriasis to interleukin-10 is associated with suppression of cutaneous type 1 inflammation, downregulation of the epidermal interleukin-8/CXCR2 pathway and normalization of keratinocyte maturation. J. investigative dermatology 116 (2), 319–329. 10.1046/j.1523-1747.2001.01248.x 11180010

[B110] Ren D. X. Cheng G. Li T. Wang Q. (2015). Determination of paeoniflorin content in Paeonia lactiflora using ultra-high-performance liquid chromatography. Chem. Eng. Manag. (22), 222–223.

[B111] Ren T. T. Liu R. Li J. Ma J. M. (2023). Primary Sjogren's syndrome misdiagnosed as Mikulicz's disease: a case report. Bmc Ophthalmol. 23 (1), 336. 10.1186/s12886-023-03090-1 37501055 PMC10375769

[B112] Santana F. P. R. Pinheiro N. M. Mernak M. I. B. Righetti R. F. Martins M. A. Lago J. H. G. (2016). Evidences of herbal medicine-derived natural products effects in inflammatory lung diseases. Mediat. Inflamm. 14, 2348968. 10.1155/2016/2348968 PMC4942669 27445433

[B113] Scheau C. Badarau I. A. Mihai L. G. Scheau A. E. Costache D. O. Constantin C. (2020). Cannabinoids in the pathophysiology of skin inflammation. Molecules 25 (3), 652. 10.3390/molecules25030652 32033005 PMC7037408

[B114] Shao Y. X. Qi X. M. Xu X. X. Wang K. Wu Y. G. Xia L. L. (2016). TGP attenuates endoplasmic reticulum stress and regulates the expression of thioredoxin-interacting protein in the kidneys of diabetic rats. Biosci. Trends 10 (6), 489–495. 10.5582/bst.2016.01188 28025459

[B115] Shen Y. Zhu L. Lin F. Yang H. (2021). Mechanism of total glucosides of paeony in the treatment of atopic dermatitis in mice. Chin. J. Clin. Pharmacol. 37 (20), 2842–2846.

[B116] Shi D. M. Ma A. D. Zheng H. Y. Huo G. Yan H. X. Fu H. J. (2014). Paeoniflorin inhibits the maturation and immunostimulatory function of allergen-induced murine dendritic cells. Int. Immunopharmacol. 19 (2), 221–232. 10.1016/j.intimp.2014.02.001 24534772

[B117] Shi D. M. Wang Q. Zheng H. L. Li D. M. Shen Y. N. Fu H. J. (2016). Paeoniflorin suppresses IL-6/Stat3 pathway via upregulation of Socs3 in dendritic cells in response to 1-chloro-2,4-dinitrobenze. Int. Immunopharmacol. 38, 45–53. 10.1016/j.intimp.2016.05.013 27236299

[B118] Shin S. H. Kim H. Y. Yoon H. S. Park W. J. Adams D. R. Pyne N. J. (2020). A novel selective sphingosine kinase 2 inhibitor, HWG-35d, ameliorates the severity of imiquimod-induced psoriasis model by blocking Th17 differentiation of naive CD4 T lymphocytes. Int. J. Mol. Sci. 21 (21), 8371. 10.3390/ijms21218371 33171607 PMC7664669

[B119] Shu Y. Liu X. Y. Luo Y. Y. Luo Y. Q. Zhou B. (2021). The clinical efficacy of total glucosides of paeony in the treatment of children with atopic dermatitis. J. Chin. Physician 23 (12), 1881–1883.

[B120] Sun Y. Zhang J. Huo R. F. Zhai T. H. Li H. D. Wu P. R. (2015). Paeoniflorin inhibits skin lesions in imiquimod-induced psoriasis-like mice by downregulating inflammation. Int. Immunopharmacol. 24 (2), 392–399. 10.1016/j.intimp.2014.12.032 25576402

[B121] Ta G. H. Weng C.-F. Leong M. K. (2021). *In silico* prediction of skin sensitization: *quo vadis*? Front. Pharmacol. 12, 655771. 10.3389/fphar.2021.655771 34017255 PMC8129647

[B122] Tang Y. J. Xu W. W. Liu X. M. Zhang R. Z. Xu C. X. Xu B. (2014). Self-control study of combination treatment of 308 nm excimer laser and calcipotriene ointment on stable psoriasis vulgaris. Int. J. Clin. Exp. Med. 7 (9), 2844–2850. 25356147 PMC4211797

[B123] Tian Y. Q. Zhang S. P. Zhang K. L. Cao D. Zheng Y. J. Liu P. (2022). Paeoniflorin ameliorates colonic fibrosis in rats with postinfectious irritable bowel syndrome by inhibiting the leptin/LepRb pathway. Evidence-Based Complementary Altern. Med. 13, 6010858. 10.1155/2022/6010858 PMC9550452 36225193

[B124] Tu J. J. Guo Y. W. Hong W. M. Fang Y. L. Han D. F. Zhang P. Y. (2019). The regulatory effects of paeoniflorin and its derivative paeoniflorin-6′-O-benzene sulfonate CP-25 on inflammation and immune diseases. Front. Pharmacol. 10, 57. 10.3389/fphar.2019.00057 30804784 PMC6370653

[B125] Türsen Ü. Türsen B. Lotti T. (2020). Coronavirus-days in dermatology. Dermatol. Ther. 33 (4), e13438. 10.1111/dth.13438 32307810 PMC7235478

[B126] Ugarte-Gil M. F. Alarcón G. S. (2014). Systemic lupus erythematosus: a therapeutic challenge for the XXI century. Clin. Rheumatol. 33 (4), 441–450. 10.1007/s10067-014-2531-4 24577816

[B127] Valdez M. A. Isamah N. Northway R. M. (2015). Dermatologic manifestations of systemic diseases. Prim. Care 42 (4), 607–630. 10.1016/j.pop.2015.08.003 26612375

[B128] Viatchenko-Karpinski V. Kong L. W. Weng H. R. (2023). Deficient AMPK activity contributes to hyperexcitability in peripheral nociceptive sensory neurons and thermal hyperalgesia in lupus mice. Plos One 18 (7), e0288356. 10.1371/journal.pone.0288356 37440542 PMC10343046

[B129] Voiculescu V. M. Lupu M. Papagheorghe L. Giurcaneanu C. Micu E. (2014). Psoriasis and Metabolic Syndrome--scientific evidence and therapeutic implications. J. Med. life 7 (4), 468–471. 25713604 PMC4316120

[B130] Wang C. Yuan J. Wu H. X. Chang Y. Wang Q. T. Wu Y. J. (2013). Paeoniflorin inhibits inflammatory responses in mice with allergic contact dermatitis by regulating the balance between inflammatory and anti-inflammatory cytokines. Inflamm. Res. 62 (12), 1035–1044. 10.1007/s00011-013-0662-8 24096935

[B131] Wang C. Yuan J. Wu H. X. Chang Y. Wang Q. T. Wu Y. J. (2015). Total glucosides of paeony inhibit the inflammatory responses of mice with allergic contact dermatitis by restoring the balanced secretion of pro-/anti-inflammatory cytokines. Int. Immunopharmacol. 24 (2), 325–334. 10.1016/j.intimp.2014.12.026 25556068

[B132] Wang G. Liu Y. F. (2004). Traditional Chinese medicine is effective and safe in the treatment of psoriasis. Int. J. Dermatology 43 (7), 552. 10.1111/j.1365-4632.2004.02181.x 15230903

[B133] Wang J. X. (2018). Editorial for biomimetic nanoparticles for drug delivery. Acta Pharm. Sin. B 8 (1), 2–3. 10.1016/j.apsb.2018.01.003 29872617 PMC5985695

[B134] Wang M. J. Wang Z. Y. Liu Y. Wang L. Wang X. M. Jiang P. (2022). The effectiveness and safety of total glucosides of paeony in systemic lupus erythematosus: a systematic review and meta-analysis. Medicine 101 (50), e32029. 10.1097/md.0000000000032029 36550839 PMC9771270

[B135] Wang Y. N. Zhang H. Du G. H. Wang Y. F. Cao T. Y. Luo Q. Q. (2016). Total glucosides of paeony (TGP) inhibits the production of inflammatory cytokines in oral lichen planus by suppressing the NF-κB signaling pathway. Int. Immunopharmacol. 36, 67–72. 10.1016/j.intimp.2016.04.010 27107800

[B136] Wang Z. Zhang G. Z. Zhang H. M. Li L. L. (2023). Xiaoyin Jiedu Granules may alleviate psoriasis-like skin diseases in mice by regulating sphingosine 1-phosphate receptor expression and reducing Th17 cells. Heliyon 9 (8), e19109. 10.1016/j.heliyon.2023.e19109 37636348 PMC10448460

[B137] Wang Z. X. Wang L. Z. (2021). Effects of total glucosides of paeony on myocardial oxidative stress andapoptosis in diabetic rats. Drug Eval. Res. 44 (03), 498–503.

[B138] Wei C. C. You F. T. Mei L. Y. Jian S. Qiang C. Y. (2013). Total glucosides of paeony prevents juxta-articular bone loss in experimental arthritis. Bmc Complementary Altern. Med. 13, 186. 10.1186/1472-6882-13-186 PMC3728075 23870279

[B139] Weidinger S. Novak N. (2016). Atopic dermatitis. Lancet 387 (10023), 1109–1122. 10.1016/s0140-6736(15)00149-x 26377142

[B140] Wong C. L. Ghassabian S. Smith M. T. Lam A. L. (2015). *In vitro* methods for hazard assessment of industrial chemicals - opportunities and challenges. Front. Pharmacol. 6, 94. 10.3389/fphar.2015.00094 25999858 PMC4419653

[B141] Wong R. S. Murphy A. Lira M. Sichmann M. G. D. Kim A. R. Saechee V. D. (2023). Microneedling with a novel, n-3-PUFA-rich formulation accelerates inflammation resolution to improve skin recovery outcomes in adults with healthy skin. Dermatology Ther. 13 (12), 3057–3069. 10.1007/s13555-023-01049-0 PMC10689607 37833618

[B142] Wu G. Wang Q. Lu W. Xiong F. Gao L. Bian H. (2021). Investigation on the mechanism of total glucosides of paeony on the treatment of sjogren's syndrome based on the regulation of RORgammat,Foxp3 and their mRNA in submandibular glands of NOD mice. Nat. Prod. Res. Dev. 33 (3), 462–467.

[B143] Wu R. H. Ma Y. H. (2023). Clinical effect of compound phellodendron gargle with total glucosides of paeony capsule in the treatment of erosive oral lichen planus. J. Pharm. Pract. Serv. 41 (01), 56–58.

[B144] Wu Y. C. Jiang Y. Y. Zhang L. Zhou J. Yu Y. Zhang S. Q. (2019). Green and efficient extraction of total glucosides from Paeonia lactiflora Pall. 'Zhongjiang' by subcritical water extraction combined with macroporous resin enrichment. Industrial Crops Prod. 141, 111699. 10.1016/j.indcrop.2019.111699

[B145] Wu Z. Wu L. J. Li L. H. Tashiro S. Onodera S. Ikejima T. (2004). p53-mediated cell cycle arrest and apoptosis induced by shikonin via a caspase-9-dependent mechanism in human malignant melanoma A375-S2 cells. J. Pharmacol. Sci. 94 (2), 166–176. 10.1254/jphs.94.166 14978355

[B146] Xiao B. H. (2019). Effects of paeoniflorin on the proliferation activity, the expression of inflammatory cytokine and chemokines of human melanocytes PIG3V induced by hydrogen peroxide and the study of NF-kappa B pathway China medical university.

[B147] Xiao X. J. Xue P. W. Shi Y. Z. Yao J. P. Cao W. Zhang L. X. (2023). The efficacy and safety of high-dose nonsedating antihistamines in chronic spontaneous urticaria: a systematic review and meta-analysis of randomized clinical trials. Bmc Pharmacol. and Toxicol. 24 (1), 23. 10.1186/s40360-023-00665-y PMC10080829 37024900

[B148] Xie P. Y. Cui L. L. Shan Y. Kang W. Y. (2017). Antithrombotic effect and mechanism of Radix paeoniae rubra. Biomed Res. Int. 9, 9475074. 10.1155/2017/9475074 PMC5337344 28299338

[B149] Xie W. Zhang C. Wang T. Wang J. S. Fu F. H. (2023a). Effects of natural products on skin inflammation caused by abnormal hormones secreted by the adrenal gland. Front. Pharmacol. 14, 1156271. 10.3389/fphar.2023.1156271 37205913 PMC10188947

[B150] Xie Y. H. Mei X. Y. Shi W. M. (2023b). Kaempferol promotes melanogenesis and reduces oxidative stress in PIG1 normal human skin melanocytes. J. Cell. Mol. Med. 27 (7), 982–990. 10.1111/jcmm.17711 36924030 PMC10064034

[B151] Xie Y. X. Deng Q. Y. Guo M. L. Li X. L. Xian D. H. Zhong J. Q. (2023c). Proanthocyanidins: a novel approach to Henoch-Schonlein purpura through balancing immunity and arresting oxidative stress via TLR4/MyD88/NF-κB signaling pathway (Review). Exp. Ther. Med. 25 (6), 300. 10.3892/etm.2023.11999 37229322 PMC10203752

[B152] Xu R. Peng J. E. Ma Z. Xie K. L. Li M. J. Wang Q. (2023). Prolonged administration of total glucosides of paeony improves intestinal immune imbalance and epithelial barrier damage in collagen-induced arthritis rats based on metabolomics-network pharmacology integrated analysis. Front. Pharmacol. 14, 1187797. 10.3389/fphar.2023.1187797 38026929 PMC10679728

[B153] Xu X. X. Qi X. M. Zhang W. Zhang C. Q. Wu X. X. Wu Y. G. (2014). Effects of total glucosides of paeony on immune regulatory toll-like receptors TLR2 and 4 in the kidney from diabetic rats. Phytomedicine 21 (6), 815–823. 10.1016/j.phymed.2013.12.003 24462407

[B154] Xu Y. He H. Li P. Liu H. W. (2022). Paeoniflorin inhibits proliferation and promotes autophagy and apoptosis of sweat gland cells. Exp. Ther. Med. 23 (1), 53. 10.3892/etm.2021.10975 34934430 PMC8652401

[B155] Yan X. Zhang B. (2016). Influence of total glucosides paeony on the expression of IFN-gammaand IL-10 in peripheral blood of patients with oral lichen planus. J. Oral Sci. Res. 32 (5), 534–537.

[B156] Yang D. Q. You L. P. Song P. H. Zhang L. X. Bai Y. P. (2012). A randomized controlled trial comparing total glucosides of paeony capsule and compound glycyrrhizin tablet for alopecia areata. Chin. J. Integr. Med. 18 (8), 621–625. 10.1007/s11655-012-1173-0 22855038

[B157] Yang D. Q. Zheng J. W. Zhang Y. M. Jin Y. L. Gan C. N. Bai Y. P. (2013). Total glucosides of paeony capsule plus compound glycyrrhizin tablets for the treatment of severe alopecia areata in children: a randomized controlled trial. Evidence-Based Complementary Altern. Med. 5, 378219. 10.1155/2013/378219 PMC3800570 24204391

[B158] Yang K. L. Zeng L. T. Long Z. Y. He Q. Xiang W. Ge A. Q. (2023). Efficacy and safety of total glucosides of paeony in the treatment of 5 types of inflammatory arthritis: a systematic review and meta-analysis. Pharmacol. Res. 195, 106842. 10.1016/j.phrs.2023.106842 37402434

[B159] Yang Y. Chen H. Ma R. Li G. Men J. (2015). Expression of STAT3 and IL-4 mRNA in peripheral blood of patients with psoriasis vulgaris before and after the treatment with total glucosides of paeony. J. Clin. Dermatology 44 (7), 423–426.

[B160] Yao S. W. Zhong Y. Q. Cai Y. J. Chen H. Xiang X. W. Zhou Y. F. (2024). Improvement mechanism of lipid metabolism and gut microbiota in obese mice with *Thunnus albacares* eggs yolk glycoprotein. J. Funct. Foods 114, 106057. 10.1016/j.jff.2024.106057

[B161] Ye R. Nie L. P. Hu X. P. Huang X. T. Jiang X. J. (2013). Clinical effect and relevant experimental study in patients with sporadic vitiligo by the combination of total glucosides of paeony and pimecrolimus. Chin. J. Dermatovenereology Integr. Traditional West. Med. 12 (03), 155–157.

[B162] Yin Y. Sun Y. Gu L. Y. Zheng W. Gong F. Y. Wu X. X. (2011). Jaceosidin inhibits contact hypersensitivity in mice via down-regulating IFN-γ/STAT1/T-bet signaling in T cells. Eur. J. Pharmacol. 651 (1-3), 205–211. 10.1016/j.ejphar.2010.10.068 21093428

[B163] Yu C. Fan X. L. Li Z. L. Liu X. M. Wang G. W. (2017a). Efficacy and safety of total glucosides of paeony combined with acitretin in the treatment of moderate-to-severe plaque psoriasis: a double-blind, randomised, placebo-controlled trial. Eur. J. Dermatology 27 (2), 150–154. 10.1684/ejd.2016.2946 28400341

[B164] Yu C. Li Z. L. Liu X. M. Wang G. (2015). Clinical efficacy of acitretin combined with total glucosides of white paeony for the treatment of plaque psoriasis. J. Clin. Med. 44 (12), 763–766. 10.16761/j.cnki.1000-4963.2015.12.020

[B165] Yu J. H. Xiao Z. C. Zhao R. Z. Lu C. J. Zhang Y. M. (2017b). Paeoniflorin suppressed IL-22 via p38 MAPK pathway and exerts anti-psoriatic effect. Life Sci. 180, 17–22. 10.1016/j.lfs.2017.04.019 28456711

[B166] Yu X. J. Li C. Y. Dai H. Y. Cai D. X. Wang K. Y. Xu Y. H. (2007). Expression and localization of the activated mitogen-activated protein kinase in lesional psoriatic skin. Exp. Mol. Pathology 83 (3), 413–418. 10.1016/j.yexmp.2007.05.002 17599830

[B167] Yuan J. P. Lu Y. S. Wang H. X. Feng Y. X. Jiang S. B. Gao X. H. (2020). Paeoniflorin resists H_2_O_2_-induced oxidative stress in melanocytes by *JNK/Nrf2/HO-1* pathway. Front. Pharmacol. 11, 536. 10.3389/fphar.2020.00536 32410998 PMC7198857

[B168] Yuan J. P. Zhu P. Y. Sun Y. Z. Lu Y. S. Qi R. Q. Chen H. D. (2024). Paeoniflorin regulates RhoA/ROCK1 and Nrf2 pathways in PDLIM1-dependent or independent manners in oxidative stressed melanocytes. Archives Dermatological Res. 316 (7), 401. 10.1007/s00403-024-03154-2 38878083

[B169] Zhang A. P. Chen S. L. Lin R. Y. (2023a). Combined use of total glucosides of paeony and hydroxychloroquine in primary Sjögren's syndrome: a systematic review. Immun. Inflamm. Dis. 11 (10), e1044. 10.1002/iid3.1044 37904705 PMC10587734

[B170] Zhang B. X. Wang J. Zhao G. D. Lin M. Lang Y. Zhang D. C. (2020). Apigenin protects human melanocytes against oxidative damage by activation of the Nrf2 pathway. Cell Stress and Chaperones 25 (2), 277–285. 10.1007/s12192-020-01071-7 31953635 PMC7058778

[B171] Zhang L. C. Chai R. R. Tai Z. G. Miao F. Z. Shi X. W. Chen Z. J. (2024a). Noval advance of histone modification in inflammatory skin diseases and related treatment methods. Front. Immunol. 14, 1286776. 10.3389/fimmu.2023.1286776 38235133 PMC10792063

[B172] Zhang L. H. Guo H. Zhang X. G. Wang L. Wei F. Zhao Y. K. (2024b). Small nucleolar RNA Snora73 promotes psoriasis progression by sponging miR-3074-5p and regulating PBX1 expression. Funct. and Integr. Genomics 24 (1), 15. 10.1007/s10142-024-01300-7 PMC10799104 38240925

[B173] Zhang L. L. Wei W. (2020). Anti-inflammatory and immunoregulatory effects of paeoniflorin and total glucosides of paeony. Pharmacol. and Ther. 207, 107452. 10.1016/j.pharmthera.2019.107452 31836457

[B174] Zhang T. Cao S. H. Yuan H. Park S. M. (2021a). Alleviation of androgenetic alopecia with aqueous Paeonia lactiflora and poria cocos extract intake through suppressing the steroid hormone and inflammatory pathway. Pharmaceuticals 14 (11), 1128. 10.3390/ph14111128 34832910 PMC8621879

[B175] Zhang T. Z. Yang Z. C. Yang S. H. Du J. Wang S. M. (2015). Immunoregulatory effects of paeoniflorin exerts anti-asthmatic effects via modulation of the Th1/Th2 equilibrium. Inflammation 38 (6), 2017–2025. 10.1007/s10753-015-0182-5 25971794

[B176] Zhang X. J. Li Z. Leung W. M. Liu L. Xu H. X. Bian Z. X. (2008). The analgesic effect of paeoniflorin on neonatal maternal separation-induced visceral hyperalgesia in rats. J. Pain 9 (6), 497–505. 10.1016/j.jpain.2007.12.009 18387856

[B177] Zhang Y. (2015). Influence of total glucvosides of paeony on skin tissue and peripheral blood mRNA expression of VEGF mRNA in BALB/c mice psoriasis. Southern Medical University. Chinese dissertation

[B178] Zhang Y. Zhang C. Y. Yuan Y. Y. Shi J. (2023b). Clinical research on the therapeutic effects of combining mycophenolate mofetil, glucocorticoids and total glucosides of paeony capsules in the treatment of systemic lupus erythematosus. J. Med. Forum 44 (19), 98–101+105.

[B179] Zhang Y. Zhou X. Sun L. D. (2021b). Effect of total glucosides of paeony on imiquimod-induced psoriatic skin lesions by regulating VEGF. Clin. Cosmet. Investigational Dermatology 14, 1889–1897. 10.2147/ccid.S339627 PMC8714466 34992404

[B180] Zhang Z. H. Jia Y. Tao L. Y. Liu X. D. Han Y. Wang X. (2022a). Clinical evaluation of dexamethasone plus gentamycin mouthwash use in combination with total glucosides of paeony for treatment of oral lichen planus without fungal infection: a comparative study with long-term follow-up. J. Clin. Med. 11 (23), 7004. 10.3390/jcm11237004 36498580 PMC9739003

[B181] Zhang Z. H. Zhang Y. Zhao Z. F. Li P. Chen D. Y. Wang W. (2022b). Paeoniflorin drives the immunomodulatory effects of mesenchymal stem cells by regulating Th1/Th2 cytokines in oral lichen planus. Sci. Rep. 12 (1), 18678. 10.1038/s41598-022-23158-0 36333421 PMC9636377

[B182] Zhao J. X. Di T. T. Wang Y. Wang Y. Liu X. Liang D. Y. (2016). Paeoniflorin inhibits imiquimod-induced psoriasis in mice by regulating Th17 cell response and cytokine secretion. Eur. J. Pharmacol. 772, 131–143. 10.1016/j.ejphar.2015.12.040 26738780

[B183] Zhao M. Liang G. P. Tang M. N. Luo S. Y. Zhang J. Cheng W. J. (2012). Total glucosides of paeony induces regulatory CD4^+^CD25^+^T cells by increasing Foxp3 demethylation in lupus CD4^+^ T cells. Clin. Immunol. 143 (2), 180–187. 10.1016/j.clim.2012.02.002 22406048

[B184] Zhao M. N. Peng N. Zhou Y. B. Qu Y. Cao M. Zou Q. H. (2024). The immunoregulatory effects of total glucosides of paeony in autoimmune diseases. J. Leukoc. Biol., qiae095. 10.1093/jleuko/qiae095 38626175

[B185] Zhao X. T. Li R. H. Qiao M. Yan J. J. Sun Q. (2018a). MiR-548a-3p promotes keratinocyte proliferation targeting PPP3R1 after being induced by IL-22. Inflammation 41 (2), 496–504. 10.1007/s10753-017-0705-3 29181737

[B186] Zhao Y. M. Xie Y. X. Li X. L. Song J. Guo M. L. Xian D. H. (2022). The protective effect of proanthocyanidins on the psoriasis-like cell models via PI3K/AKT and HO-1. Redox Rep. 27 (1), 200–211. 10.1080/13510002.2022.2123841 36178125 PMC9542435

[B187] Zhao Z. F. Han Y. Zhang Z. H. Li W. W. Ji X. L. Liu X. D. (2018b). Total glucosides of paeony improves the immunomodulatory capacity of MSCs partially via the miR-124/STAT3 pathway in oral lichen planus. Biomed. and Pharmacother. 105, 151–158. 10.1016/j.biopha.2018.05.076 29852392

[B188] Zhou L. L. Cao T. Y. Wang Y. F. Yao H. Du G. H. Tian Z. (2016a). Clinical observation on the treatment of oral lichen planus with total glucosides of paeony capsule combined with corticosteroids. Int. Immunopharmacol. 36, 106–110. 10.1016/j.intimp.2016.03.035 27129091

[B189] Zhou P. Yang X. D. Jia X. Y. Yu J. Asenso J. Xiao F. (2016b). Effect of 6'-acetylpaeoniflorin on dinitrochlorobenzene-induced allergic contact dermatitis in BALB/c mice. Immunol. Res. 64 (4), 857–868. 10.1007/s12026-016-8788-8 26798038

[B190] Zhou Z. Lin J. P. Huo R. F. Huang W. K. Zhang J. Wang L. (2012). Total glucosides of paeony attenuated functional maturation of dendritic cells via blocking TLR4/5 signaling *in vivo* . Int. Immunopharmacol. 14 (3), 275–282. 10.1016/j.intimp.2012.07.012 22846756

[B191] Zhu Y. P. Wang S. Q. Lin F. Q. Li Q. Xu A. (2014). The therapeutic effects of EGCG on vitiligo. Fitoterapia 99, 243–251. 10.1016/j.fitote.2014.08.007 25128425

[B192] Zuo Z.-Y. Zhan S.-Y. Huang X. Ding B.-Y. Liu Y.-Q. Ruan Y.-E. (2017). Research progress of pharmacokinetics and pharmacodynamics of total glucosides of peony in hepatoprotective effects. China J. Chin. materia medica 42 (20), 3860–3865. 10.19540/j.cnki.cjcmm.2017.0153 29243418

